# Age-related retinal degeneration resulting from the deletion of Shp2 tyrosine phosphatase in photoreceptor neurons

**DOI:** 10.1038/s41419-024-06924-y

**Published:** 2024-08-08

**Authors:** Ammaji Rajala, Rahul Rajala, Mohd A. Bhat, Mark Eminhizer, Jeff Hao, Jianhai Du, Raju V. S. Rajala

**Affiliations:** 1 Department of Ophthalmology, University of Oklahoma Health Sciences Center, Oklahoma City, OK 73104 USA; 2 Dean McGee Eye Institute, Oklahoma City, OK 73104 USA; 3 Oklahoma Medical Research Foundation, Oklahoma City, OK 73104 USA; 4 Department of Cell Biology, University of Oklahoma Health Sciences Center, Oklahoma City, OK 73104 USA; 5 Departments of Ophthalmology and Visual Sciences and Biochemistry and Molecular Medicine, West Virginia University, Morgantown, WV 26505 USA; 6 Department of Biochemistry and Physiology, University of Oklahoma Health Sciences Center, Oklahoma City, OK 73104 USA

**Keywords:** Neurotrophic factors, Ageing

## Abstract

Shp2, a critical SH2-domain-containing tyrosine phosphatase, is essential for cellular regulation and implicated in metabolic disruptions, obesity, diabetes, Noonan syndrome, LEOPARD syndrome, and cancers. This study focuses on Shp2 in rod photoreceptor cells, revealing its enrichment, particularly in rods. Deletion of Shp2 in rods leads to age-dependent photoreceptor degeneration. Shp2 targets occludin (OCLN), a tight junction protein, and its deletion reduces OCLN expression in the retina and retinal pigment epithelium (RPE). The isolation of actively translating mRNAs from rods lacking Shp2, followed by RNA sequencing, reveals alterations in cell cycle regulation. Additionally, altered retinal metabolism is observed in retinal cells lacking Shp2. Our studies indicate that Shp2 is crucial for maintaining the structure and function of photoreceptors.

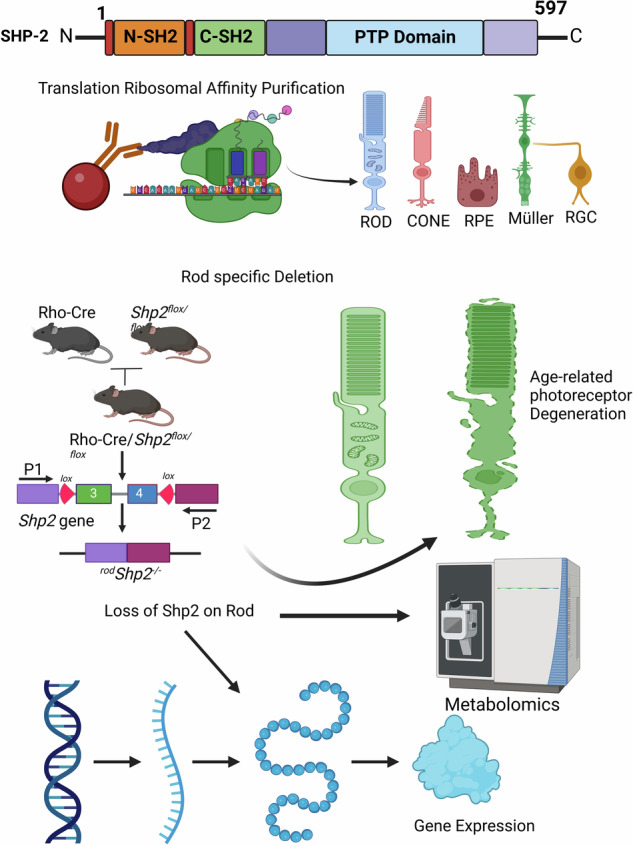

## Introduction

Src homology region 2 (SH2)-containing protein tyrosine phosphatase 2 (Shp2), is a non-receptor phosphotyrosine phosphatase, encoded by the *ptpn11gene* [1, 2]. Shp2 is a cytoplasmic phosphatase ubiquitously expressed in cells and various tissues [2]. Shp2 regulates both receptor tyrosine kinase and non-receptor tyrosine kinase-regulated pathways [2–4]. Shp2 activity plays an important role in regulating tumorigenesis, development, and metabolic diseases [5, 6]. An earlier study shows that Shp2 protein expression is absent from the photoreceptor cells and loss of Shp2 in the retina results in photoreceptor degeneration and suggests that Shp2 may be indirectly required for photoreceptor cell survival, a non-cell-autonomous function of Shp2 [7]. Interestingly, we previously reported that the Shp2 substrate, Grb2-associated binding protein 1 (Gab1) is predominantly expressed in photoreceptor cells [8]. These observations raise several questions, which include 1) whether Shp2 is expressed in photoreceptor cells, and 2) how photoreceptor degeneration occurs in the retina-specific deletion of Shp2, which we addressed in this study. We have employed state-of-the-art biochemical, bioinformatics, and functional techniques to demonstrate that Shp2 is expressed in photoreceptors and rod-cell-specific deletion of Shp2 results in photoreceptor degeneration. Our studies suggest that Shp2 is essential for the maintenance of the structure, function, and viability of photoreceptor cells.

## Results

### Characterization of Shp2 in rod photoreceptors

**Fig. 1:**
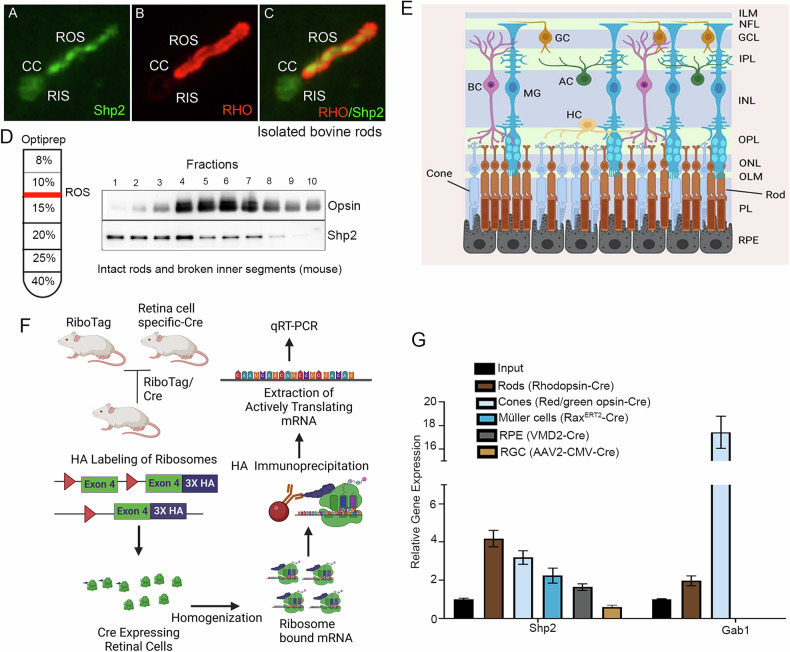
Expression of Shp2 in various retinal cells. Isolated bovine rods were immunostained with Shp2 (green) and rhodopsin (red) antibodies (**A**–**C**). ROS, rod outer segments, RIS, rod inner segments, CC, connecting cilium. Retina lysate was subjected to Opti-Prep (8–40%) density gradient centrifugation and the fractions were immunoblotted with Shp2 and opsin (rhodopsin) (**D**). Cross section of the retina displaying all the retinal cells (**E**). Translating Ribosome Affinity Purification. Floxed Rpl22 mice were mated with rod-Cre (**F**), cone-Cre, Müller cell-Cre, RPE-Cre, and RGC-Cre individually and the ribosomes were immuno-affinity purified with HA antibody. The bound mRNA associated with ribosomes was isolated (**F**). Equal amounts of mRNA from various retinal cells were quantified using Shp2 and Gab1 primers, and the data was normalized to the housekeeping gene Rpl37 (**G**). Input represents the total retinal RNA. Data are mean ± *SEM* (*n* = *3*). Panels (**E**, **F**) were created with BioRender.com. Note: The opsin blot shown in (**D**) was previously used in another study [41]. The same samples were used to examine Shp2 expression.

To determine whether Shp2 is expressed in rod photoreceptor cells, we have isolated bovine rods, which contain both rod outer and inner segments co-immunolabelled with rhodopsin and Shp2 antibodies. We found the expression of Shp2 in both the rod outer and inner segments whereas the rod outer segment marker, rhodopsin exclusively stained the outer segments (Fig. 1A–C). To validate further the expression of Shp2 in rod photoreceptors, we isolated intact photoreceptors on Opti-prep density gradient centrifugation, and the fractions were immunoblotted with Shp2 and rhodopsin antibodies. Our results further show that Shp2 co-eluted with rhodopsin suggesting the expression of Shp2 in rod photoreceptor cells (Fig. 1D).

Retina is a heterogeneous tissue composed of several cell types (Fig. 1E). To further examine the cell-type specific expression of Shp2, we employed Translating Ribosome Affinity Purification (TRAP) by breeding RiboTag mice which allows for Cre-mediated labeling of ribosomes with an HA-tagged ribosomal protein Rpl22; with mice carrying a rod specific Cre, allowing for the labeling of Rod Polysomes with HA. Immunoprecipitation of polysomes with antibody against HA results in the isolation of actively translating mRNA and quantitative real-time PCR (qRT-PCR) with gene-specific primers reveal the quantitative expression of the gene of interest (Fig. 1F). In this study, we bred RiboTag mice with rod photoreceptor-specific rhodopsin-Cre, cone photoreceptor-specific red/green opsin-Cre, retinal pigment epithelial-specific VMD2-Cre, Müller cell-specific Rax-Cre and AAV2-CMV-Cre to target retinal ganglion cells as we described earlier [9]. Isolated polysome-bound actively translating mRNA was subjected to qRT-PCR with Shp2 primers (Table 1) and normalized the expression to *Rpl37* ribosomal protein [9]. Our results indicate increased expression of Shp2 in rods followed by cone, Müller cells, and RPE compared to input (total RNA isolated from respective Cre/Rpl22 mouse retina) and ganglion cells (Fig. 1G). We previously reported that Shp2 substrate Gab1 is predominantly expressed in rod inner segments [8]. Our TRAP data indicate increased expression of Gab1 in both rods and cones but not in RPE, Müller and ganglion cells (Fig. 1G). Collectively, our data confirm the expression of Shp2 in rod photoreceptor cells.

**Table 1:** Real-time PCR primers to quantitate the expression of *Shp2, Gab1, Occludin*, and inflammatory markers.

Gene	Forward Primer	Reverse Primer
*Shp2*	CCATGGGCAGCTGAAAGAGA	TACGAGTTGTGTTGAGGGGC
*Gab1*	CTCCGGAGGAGTTTCAGAGAA	TTCGCGGACTGAAGAGGAAC
*Occludin*	TTGAAAGTCCACCTCCTTACAGA	CCGGATAAAAAGAGTAGCCTGG
*Rpl37*	CGGGACTGGTCGGATGAG	TCACGGAATCCATGTCTGAATC
*Actb* (*β-actin*)	ACTGGGACGACATGGAGAAG	GGGGTGTTGAAGGTCTCAAA
*Arg1*	GAACACGGCAGTGGCTTTAAC	TGCTTAGCTCTGTCTGCTTTGC
*Cd206*	TCTTTGCTTTCCAGTCTCC	TGACACCCAGCGGAATTTC
*Cd32*	AATCCTGCCGTTCCTACTGATC	GTGTCACCGTGTCTTCCTTGAG
*Gfap*	CCAGCTTACGGCCAACAGT	TGGTTTCATCTTGGACTTCTG
*Il-8*	CCTGCTCTGTCACCGATG	CAGGGCAAAGAACAGGTCAG
*Il-10*	ACCTGCTCCACTGCCTTGCT	GGTTGCCAAGCCTTATCGGA
*Il-1β*	TCGAGGCCTAATAGGCTCATCT	GCTGCTTCAGACACTTGCACAA
*Ifn-γ*	TGGCTCTGAAGGATTTTCATC	TCAACTGGCATAGATGTGGAAGAA
*Inos*	CATTGGAAGTGAAGCGTTTCG	CAGCTGGGCTGTACAAACCTT
*Mcp1*	AAGTGCAGAGAGCCAGACG	TCAGTGAGAGTTGGCTGGTG
*Nfh*	CACCAAGGAGTCACTGGAG	TGCTGAATAGCGTCCTGGTA
*Tnf-α*	CCTCTTCTCATTCCTGCTTGTGG	GGCCATTTGGGAACTTCTCATC

### Functional role of Shp2 in rod photoreceptor cells

**Fig. 2:**
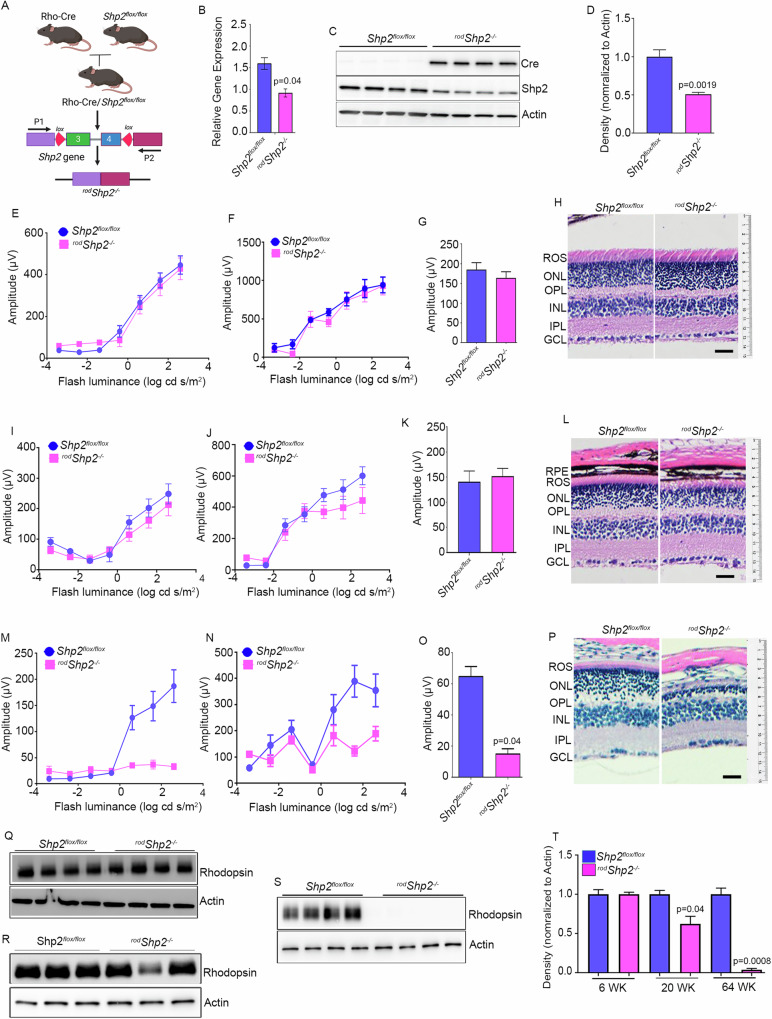
Functional and morphological characterization of conditional rod-specific Shp2 KO mice. **A** Rod-specific Shp2 KO mice (^*rod*^Shp2^*−/−*^) were generated by breeding floxed Shp2 mice (Shp2^*flox/flox*^) with mice expressing Cre-recombinase under the control of rhodopsin promoter. Quantitative real-time PCR shows the normalized Shp2 expression to actin in Shp2^*flox/flox*^ and ^*rod*^Shp2^*−/−*^ mice (**B**). Data are mean ± *SEM* (*n* = *3*). Retina lysates were prepared from one-month-old mice Shp2^*flox/flox*^ and ^*rod*^Shp2^*−/−*^ mice and immunoblotted with Cre, Shp2, and actin antibodies (**C**). Densitometric analysis of Shp2 normalized to actin (**D**). Data are mean ± *SEM* (*n* = *3)*. Functional characterization of ^*rod*^Shp2^*−/−*^ mouse retina. Scotopic a-wave (**E**, **I**, **M**), scotopic b-wave (**F**, **J**, **N**), and photopic b-wave (**G**, **K**, **O**) analyses were performed on 6-week-old (**E**–**G**), 20-week-old (**I–K**), and 64-week-old (**M**–**O**) Shp2^*flox/flox*^ and ^*rod*^Shp2^*−/−*^ mice. Data are mean ± *SEM* (*n* = 8). The ^*rod*^Shp2^*−/−*^ response was significantly lower than that of the Shp2^*flox/flox*^ retinas (*p* < 0.001). Data were analyzed by two-way ANOVA and unpaired t-test. Six-week (**H**), 20-week (**L**), and 64-week-old (**P**) Shp2^*flox/flox*^ and ^*rod*^Shp2^*−/−*^ mouse eye sections were stained with hematoxylin and eosin staining. Retinal proteins from 6-week (**Q**), 20-week (**R**), and 64-week-old (**S**) Shp2^*flox/flox*^ and ^*rod*^Shp2^*−/−*^ mice were immunoblotted with rhodopsin and actin antibodies. Densitometric analysis of Shp2 normalized to actin (**T**). Data are mean ± *SEM* (*n* = 4 for 6 and 64 weeks*; n* = *3* for 20 weeks). Panels (**A**) was created with BioRender.com. Note: We used the same actin blot in Figs. 2R, 3D.

We generated rod-specific Shp2 KO mice under the control of rhodopsin promoter [10] (Fig. 2A). Quantitative real-time PCR analysis on Shp2 KO (^*rod*^*Shp2*^−/−^) mice show a significant decrease of Shp2 expression compared to wild type (*Shp2* ^*flox/flox*^) mice (Fig. 2B). There is a lack of Shp2 antibodies which are suitable for immunofluorescence, we immunoblotted the *Shp2* ^*flox/flox*^ and ^*rod*^*Shp2*^−/−^ mouse retinas with a Shp2 antibody showing a 60% decrease of Shp2 levels in ^*rod*^*Shp2*^−/−^ mice compared to *Shp2* ^*flox/flox*^ mouse retinas, consistent with the Cre-antibody reactivity only in ^*rod*^*Shp2*^−/−^ mouse retina (Fig. 2C, D). In ^*rod*^*Shp2*^−/−^ mouse retina, the remaining 40% of Shp2 protein likely comes from other retinal cell types. At 6 weeks of age, there was no significant difference in rod scotopic a- (Fig. 2E) and b-wave (Fig. 2F) and cone photopic b-wave (Fig. 2G) amplitudes between ^*rod*^*Shp2*^−/−^ and *Shp2* ^*flox/flox*^ mice. Consistent with the function, there was no significant difference in the morphology of the retina between these two genotypes (Fig. 2H). At 20 weeks of age, ^*rod*^*Shp2*^−/−^ mice showed a significant reduction in scotopic a-wave (Fig. 2I) and b-wave (Fig. 2J) amplitudes compared to *Shp2* ^*flox/flox*^ mice. However, there was no significant difference in photopic b-wave amplitudes between *Shp2* ^*flox/flox*^ and ^*rod*^*Shp2*^−/−^ mice (Fig. 2K). At this time point, we found thinning of both rod outer segment (ROS) and outer nuclear layer (ONL) thickness in ^*rod*^*Shp2*^−/−^ mice compared to *Shp2* ^*flox/flox*^ mice (Fig. 2L). At 64 weeks, ^*rod*^*Shp2*^−/−^ mice exhibited a complete loss of scotopic a-wave (Fig. 2M), significantly reduced scotopic b-wave (Fig. 2N) and photopic b-wave amplitudes compared to *Shp2* ^*flox/flox*^ mice. Morphological analysis revealed the loss of ROS and ONL layers (Fig. 2P), indicating that Shp2 deficiency in rods leads to age-related photoreceptor degeneration. To confirm the age-related morphological changes in ^*rod*^*Shp2*^−/−^ mice, we performed immunoblotting of retinal proteins from *Shp2* ^*flox/flox*^ and ^*rod*^*Shp2*^−/−^ mice using a rhodopsin antibody. The results showed no change in rhodopsin levels between *Shp2* ^*flox/flox*^ and ^*rod*^*Shp2*^−/−^ mice at 6 weeks of age (Fig. 2Q, T). However, there was a significant decrease in rhodopsin levels at 20 weeks (Fig. 2R, T) and a complete absence of rhodopsin at 64 weeks (Fig. 2S, T) in ^*rod*^*Shp2*^−/−^ mice compared to *Shp2* ^*flox/flox*^ mice. These findings further confirm that the loss of Shp2 in rods leads to age-related photoreceptor degeneration.

### Biochemical studies on Shp2 KO mice

**Fig. 3:**
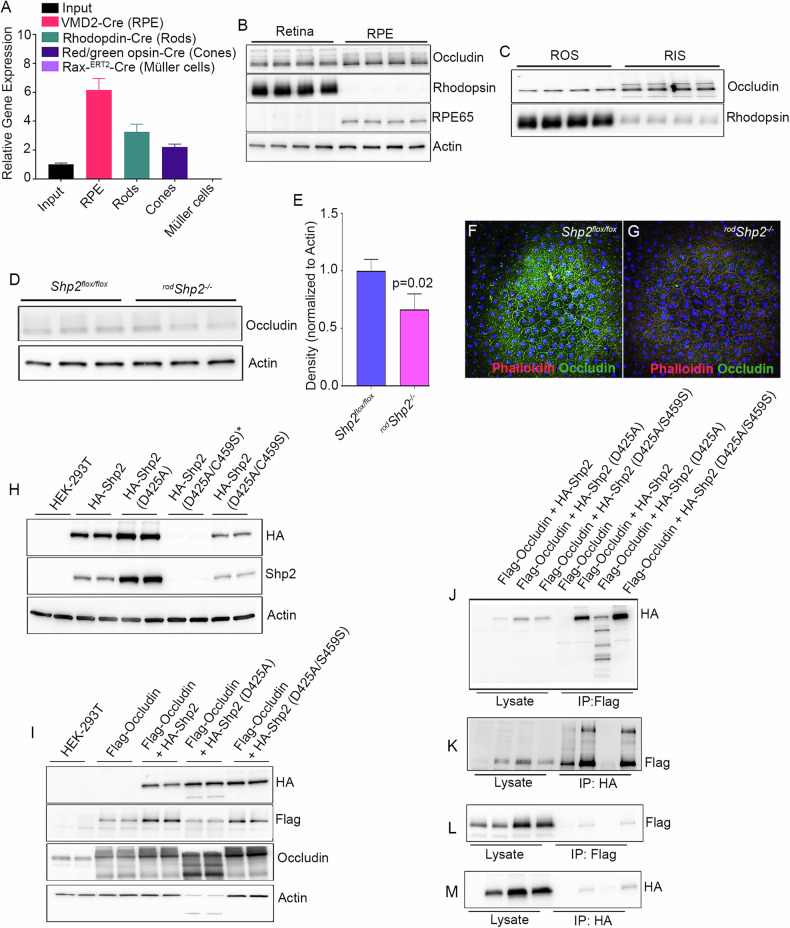
Expression of and interaction of Occludin with Shp2. TRAP isolated mRNA from input, RPE, rod, cone, and Müller cells were subjected to qRT-PCR with Occludin primers, and the data was normalized to the *Rpl37* gene (**A**). Data are mean ± *SEM* (*n* = *3)*. Retina and RPE proteins from C57Bl6 mice were immunoblotted with Occludin, rhodopsin, RPE65, and actin antibodies (**B**). Rod outer segments (ROS) and rod-enriched inner segments (RIS) were immunoblotted with Occludin and rhodopsin (**C**). Retinal proteins from Shp2^*flox/flox*^ and ^*rod*^Shp2^*−/−*^ mice were immunoblotted with Occludin and actin antibodies (**D**). Densitometric analysis of OCLN was normalized to actin (**E**). Data are mean ± *SEM* (*n* *=* *3*). RPE flat mounts from 20-week old Shp2^*flox/flox*^ (**F**) and ^*rod*^Shp2^*−/−*^ (**G**) mice were stained with Phalloidin and Occludin. Interaction between Shp2 and Occludin in a heterologous system. HEK-293T cells were transfected with WT-HA-Shp2, and mutant construct of Shp2, HA-Shp2(D425A), and HA-Shp2 (D425A/C459S) and 48 h later, proteins were immunoblotted with HA, Shp2 and actin antibodies (**H**). HEK-293T cells were transfected alone with Flag-OCLN or co-transfected with WT-HA-Shp2, and mutant construct of Shp2, HA-Shp2(D425A), and HA-Shp2 (D425A/C459S) and 48 h later, proteins were immunoblotted with HA, Flag, Occludin and actin antibodies (**I**). Reciprocal immunoprecipitations with Flag (**J**) or HA (**K**) antibodies and the interaction are examined by immunoblotting with HA (**J**, **M**), Flag (**K**, **L**) antibodies. Note: *We expressed four independent clones of HA-Shp2 (D425A/C459S) in HEK293T cells, but only two of these clones were successfully expressed.

Recent studies show that Occludin (OCLN) is a Shp2 substrate [11]. OCLN is a novel integral membrane protein localizing at tight junctions [12]. Furthermore, tight junction protein interactions and barrier function is mediated through OCLN S408 phosphorylation [13]. OCLN is also expressed in cells without tight junctions suggesting additional functions that are unrelated to tight junction formation [14]. We examined OCLN levels in TRAP samples isolated from retinal cell types show the highest expression in retinal pigment epithelium (RPE) followed by rods and cones whereas Müller cells do not express OCLN (Fig. 3A). These studies show that OCLN is not only expressed in RPE as it is required for tight junction formation but also expressed in both rod and cone photoreceptor cells.

To confirm that OCLN is expressed in the retina, we prepared retina and RPE and the protein lysates were immunoblotted with OCLN, rhodopsin, and RPE65 antibodies. The results show that rhodopsin is expressed in the retina but not in the RPE, whereas RPE65 is expressed in the RPE but not in the retina (Fig. 3B). Under these conditions, we found the expression of OCLN in both the retina and RPE (Fig. 3B), suggesting that OCLN is present in both tissues. To examine whether OCLN is expressed in rod photoreceptor cells, we isolated these cells using discontinuous sucrose density gradient centrifugation. This process yields pure outer segment membranes (ROS) and an enriched inner segment fraction (RIS), which also includes other inner retinal cells [9]. These fractions were immunoblotted with OCLN and rhodopsin antibodies, and the results demonstrate that increased OCLN is associated with RIS fractions compared to ROS; nevertheless, ROS also shows the expression of OCLN (Fig. 3C). To determine whether the loss of Shp2 in rod photoreceptor cells affects OCLN levels in the retina, we examined OCLN levels in *Shp2* ^*flox/flox*^ and ^*rod*^*Shp2*^−/−^ mouse retinas. The results show significantly reduced OCLN levels in ^*rod*^*Shp2*^−/−^ retinas compared to *Shp2* ^*flox/flox*^ retinas (Fig. 3D, E). This suggests that Shp2 plays a role in the expression or turnover of OCLN in photoreceptor cells.

Our TRAP data show increased expression of OCLN in the RPE (Fig. 3A). To examine whether the loss of Shp2 in rods affects OCLN expression in the RPE, we prepared RPE flat mounts from five-month-old *Shp2* ^*flox/flox*^ and ^*rod*^*Shp2*^−/−^ mice and stained them with OCLN and Phalloidin. The results revealed intense OCLN staining in the RPE of *Shp2* ^*flox/flox*^, whereas there was a complete loss of OCLN staining in the RPE of ^*rod*^*Shp2*^−/−^ mice (Fig. 3F, G). These findings suggest that the loss of Shp2 in rods affects the expression or turnover of OCLN in the RPE.

### Interaction of Shp2 with OCLN in a heterologous system

Recent studies show that OCLN is a Shp2 substrate [11]. Mutation of Asp to Ala in the conserved WPD loop in the protein tyrosine phosphatases (PTPs) acts as a high-affinity trap to lock their substrates [15]. Several studies have identified PTP substrates using this technology [8, 15]. Shp2 “Substrate-trapping” mutants have been used to identify Shp2 substrates [16]. We generated three Shp2 constructs (Shp2-WT, Shp2-D425A, and Shp2-D425A/C459S) with a HA-tagged epitope. Flag-tagged OCLN and HA-tagged WT and mutant Shp2 constructs were either expressed alone (Fig. 3H) or co-expressed (Fig. 3I) in HEK-293T cells. Co-expression of HA-Shp2-D425A mutant with Flag-OCLN resulted in proteolysis (Fig. 3I). Co-immunoprecipitation experiments (Fig. 3J–M) suggest that Flag-OCLN can associate with HA-Shp2-WT and HA-Shp2-D425A/C459S and vice versa (Fig. 3J, K). These observations suggest that OCLN interacts with Shp2 in vitro. We could not identify the in vivo interaction between Shp2 and OCLN, as we do not have good antibodies to study their physical interaction via immunofluorescence or co-immunoprecipitation.

### Shp2-regulated gene expression in rod photoreceptor cells

**Fig. 4:**
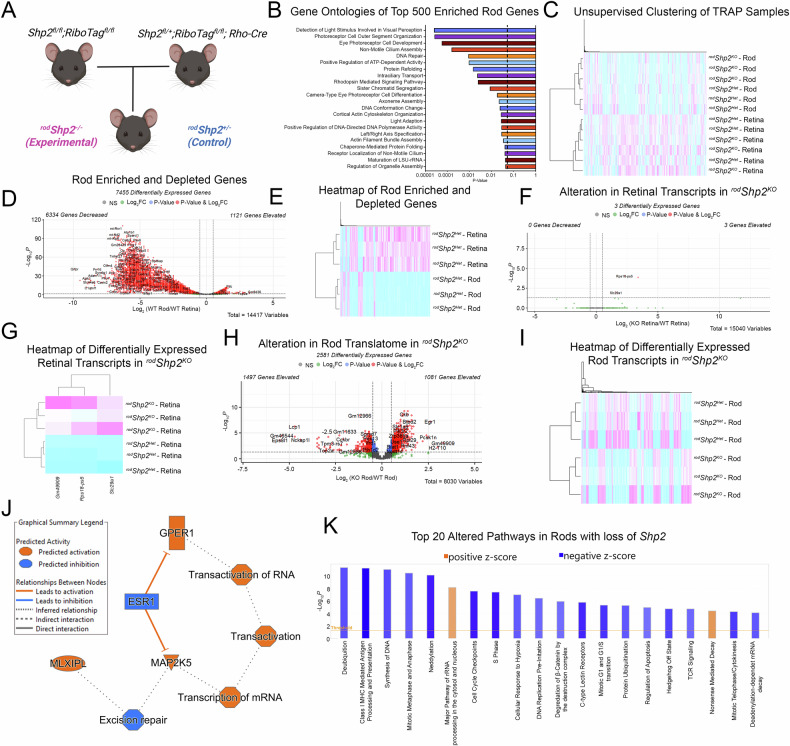
TRAP and IPA analysis of *Shp2* loss in rod photoreceptors. We employed a unique in vivo approach using TRAP. Rod-specific Shp2/Ribo-Tag mice were created by crossing homozygous Shp2 floxed mice with homozygous Ribo-Tag mice, along with rhodopsin-Cre mice. The resulting mice, with one Shp2 allele, homozygous for Ribotag, and carrying the rhodopsin Cre allele, were then bred to generate Cre-positive mice for comparative analysis: heterozygous (^*rod*^*Shp2*^*+/-*^: Control) and homozygous (^*rod*^*Shp2*^*−/−*^: KO). All experimental mice were homozygous for RiboTag (**A**). Gene Ontologies of Top 500 Rod-Enriched Transcripts (**B**). Unsupervised hierarchical clustering of TRAP and Retinal Samples (**C**). Volcano plot of differentially expressed transcripts between rod and retinal fractions (**D**). Heatmap of differentially expressed transcripts between rod and retinal fractions (**E**). Volcano plot of differentially expressed transcripts between retinal fractions of ^*rod*^*Shp2* WT and KO mice (**F**). Heatmap of significant differentially expressed transcripts between retinal fractions of ^*rod*^*Shp2* WT and KO mice (**G**). Volcano plot of differentially expressed transcripts between rod fractions of ^*rod*^*Shp2* WT and KO mice (**H**). Heatmap of significant differentially expressed transcripts between rod fractions of ^*rod*^*Shp2* WT and KO mice (**I**). Graphical Summary of IPA network analysis showing potential linchpin genes. Orange: Predicted Activation, Blue: Predicted Inhibition (**J**). Prediction of differentially affected pathways in rods mediated by loss of *Shp2*. Orange: Predicted Activation (Positive Z-Score), Blue: Predicted Inhibition (Negative Z-Score) (**K**). Panel (**A**) was created with BioRender.com.

We generated Rod-specific Shp2/ Ribo-Tag mice by first breeding homozygous Shp2 floxed mice with homozygous Ribo-Tag mice. Simultaneously, we bred homozygous Ribo-Tag mice with rhodopsin-Cre mice. Males who carried one allele of Shp2, were homozygous for Ribo-Tag, and carrying the rhodopsin Cre was then mated to females who were homozygous for both Shp2 and Ribo-Tag (Fig. 4A). As a result, Cre-positive mice who were heterozygous for (^*rod*^*Shp2*^*+/-*^: Control) were compared to Cre-positive homozygous mice (^*rod*^*Shp2*^*−/−*^: KO*)*; additionally, all experimental mice were homozygous for Ribo Tag. Polyribosome immunoprecipitation was then carried out on retinas, as described previously [9] and both input and HA-IP fractions were subjected to RNA sequencing at MedGenome Inc (Foster City, CA).

RNA sequencing resulted in reads on ~14,417 genes in our retina samples and ~11,600 genes in our rod-enriched samples. GO Analysis (http://geneontology.org/) of the Top 500 enriched genes after Rod-TRAP showed transcripts involved in processes of visual perception, photoreceptor function and development, and ciliary assembly. Further validating Rod-TRAP as a suitable method to enrich Rod Specific transcripts (Fig. 4B). Rod-TRAP transcripts were also depleted for non-Rod photoreceptor markers, as well as mitochondrial genes (Fig. 4D, E). This observation was further confirmed with unsupervised hierarchical clustering of Rod-TRAP and retinal samples, showing clustering between Rod-TRAP and retinal samples, and sub-clustering of Shp2 WT and Shp2 null Rod-Trap Samples. (Fig. 4C) A comparison between retinal mRNA samples of identified three differentially regulated genes Slc29a1, Rps18-ps5, and Gm49909. (Fig. 4F, G). To identify alterations between rod-enriched mRNA samples of ^*rod*^*Shp2*^*+/-*^ and ^*rod*^*Shp2*^*−/−*^, we removed all rod-depleted genes from our rod translatome datasets; this was done to ensure we assayed for changes occurring only with rod-specific transcripts. A comparison between rod-enriched mRNA samples of ^*rod*^*Shp2*^*+/-*^ and ^*rod*^*Shp2*^*−/−*^identified 2581 differentially regulated genes between samples (Fig. 4H, I); these genes were then used for Ingenuity Pathway Analysis (IPA).

IPA Network analysis revealed predicted activation of Mitogen-Activated Protein Kinase 5 (MAP2K5), MLX interacting protein-like (MLXIPL), and G-protein coupled estrogen receptor 1 (GPER1) (Fig. 4J). The top dysregulated pathways revealed by IPA included Sirtuin Signaling Pathway $(p < 8.72E-19),$ Nucleotide Excision Repair Enhanced Pathway $(p < 3.76E-16)$, and the Protein Ubiquitination Pathway $(p < 4.04E-17)$. To identify significantly altered pathways with consistent activation or inhibition, we subset all identified altered pathways which had a $(z-{score} > 2.0){\rm{and\; a}}{-\log }_{10}{(P}_{{Value}}) > 4$, this yielded 20 significantly altered pathways. Surprisingly, a majority of these changes were in Pathways linked to the Cell Cycle (Fig. 4K). IPA also identified the likely activation of upstream regulators, MLX interacting protein-like (Mlxipl), and serine/threonine kinase 11 (Stk11), and the likely inhibition of upstream regulators COP9 Signalosome Subunit 5 (Cops5), and protein tyrosine phosphatase receptor type R (Ptprr).

**Fig. 5:**
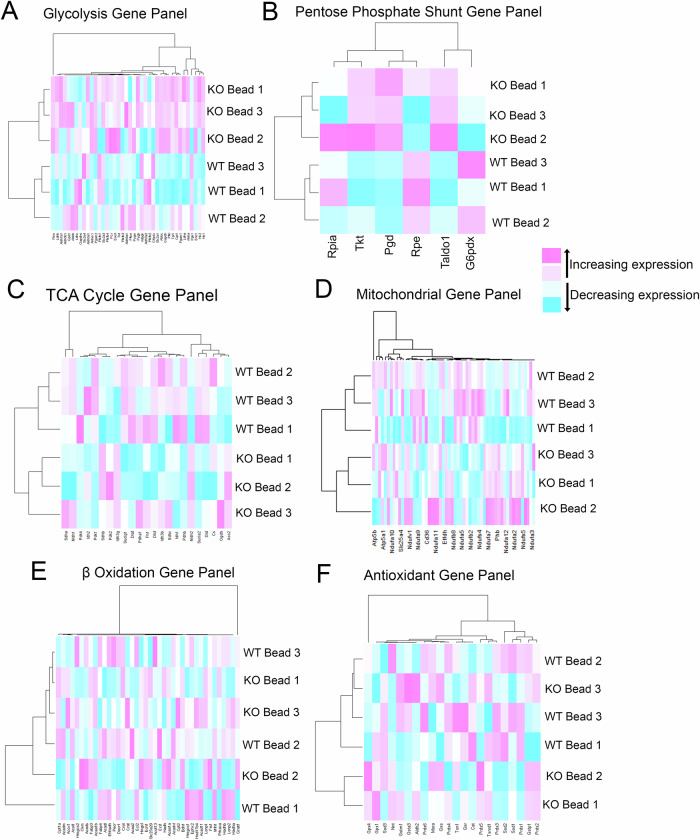
Loss of Shp2 in rods alters cellular metabolism. Unsupervised hierarchical clustering glycolytic transcripts between rod fractions of ^*rod*^*Shp2* WT and KO mice (**A**). Unsupervised hierarchical clustering pentose phosphate shunt (PPS) transcripts between rod fractions of ^*rod*^*Shp2* WT and KO mice (**B**). Unsupervised hierarchical clustering TCA Cycle transcripts between rod fractions of ^*rod*^*Shp2* WT and KO mice (**C**). Unsupervised hierarchical clustering of mitochondrial transcripts between rod fractions of ^*rod*^*Shp2* WT and KO mice (**D**). Unsupervised hierarchical clustering of fatty acid oxidation transcripts between rod fractions of ^*rod*^*Shp2* WT and KO mice (**E**). Unsupervised hierarchical clustering of antioxidant transcripts between rod fractions of ^*rod*^*Shp2* WT and KO mice (**F**).

Unsupervised hierarchical clustering was also performed on the translatomes between the Rod Fractions of ^*rod*^*Shp2* WT and KO mice; using metabolic gene panels designed for Glycolysis, Pentose Phosphate Shunt (PPS), TCA Cycle, Mitochondria, Fatty Acid Oxidation (FAO) and Antioxidants (Fig. 5). The separation between ^*rod*^*Shp2* WT and KO mice could be observed in Glycolysis, PPS, TCA, and Mitochondria, but not in FAO or Antioxidant Gene Panels. This suggests an alteration in cellular metabolism; heat maps showed an increase in glycolytic and PPS gene transcripts in *Shp2* KO rods but decreased TCA gene panels in *Shp2* KO rods. Mitochondrial gene panels showed separation between ^*rod*^*Shp2* WT and KO mice but no discernable trend in overall transcripts, suggesting more stringent functional analysis of ^*rod*^*Shp2* KO rod mitochondria is needed to identify any potential functional changes.

### Altered retinal metabolism in rods lacking Shp2

**Fig. 6:**
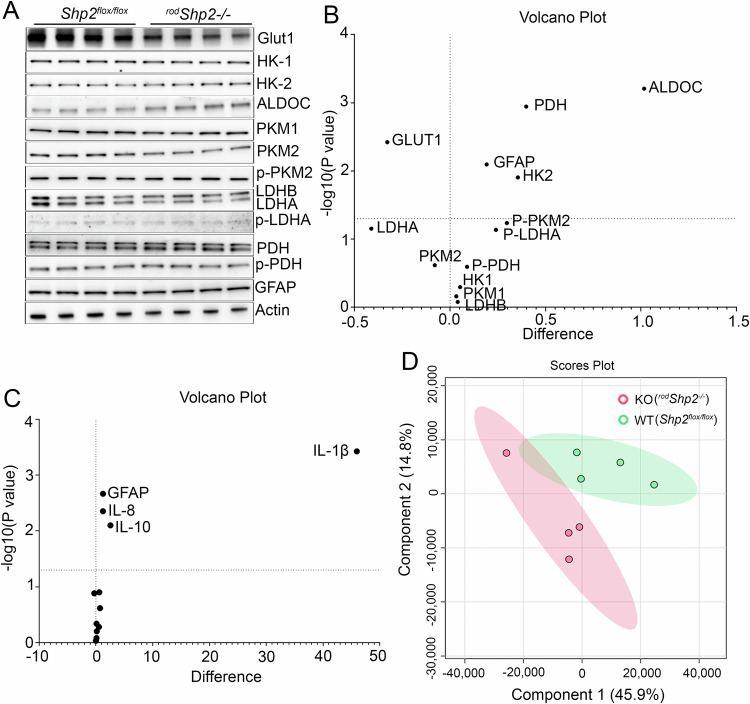
Effect of loss of Shp2 on retinal metabolism. Immunoblot analysis of 6-week-old *Shp2*^*flox/flox*^ and ^*rod*^*Shp2*^*−/−*^ mouse retinas with Glut1, HK-1, HK-2, ALDOC, PKM1, PKM2, p-PKM2, LDHB, LDHA, p-LDHA, PDH, p-PDH, GFAP and actin (**A**) antibodies. Volcano plot showing differential protein levels of selected proteins from the immunoblots of **6** **A**, horizontal line indicates changes with (*p* < 0.05) (**B**) (*n* = *4*). Quantitative real-time PCR analysis to examine the expression of genes related to inflammation. Equal amounts of retinal mRNA from three independent (*n* = *3*) *Shp2*^*flox/flox*^ and ^*rod*^*Shp2*^*−/−*^ mice were used for RT-PCR and normalized by β-actin levels. Volcano plot of differentially expressed inflammatory gene transcripts in ^*rod*^*Shp2*^*−/−*^ mice compared to *Shp2*^*flox/flox*^ mice, horizontal line indicates changes with (*p* < 0.05) (**C**). ARG1, Arginase 1; CD206, mannose receptor; CD32, cluster of differentiation 32; GFAP, glial fibrillary acidic protein; IL-8, interleukin-8; IL-10, interleukin 10; IL-1β, interleukin-1β; IFNγ, interferon gamma; iNOS, inducible nitric oxide synthase 2; MCP1, monocyte chemoattractant protein-1; NFH, neurofilament heavy chain; TNFα, tumor necrosis factor alpha. LC-MS was used to examine steady-state levels of 126 metabolites in four-independent *Shp2*^*flox/flox*^ and ^*rod*^*Shp2*^*−/−*^ mouse retinas that are representative of major pathways in metabolism, including glucose, lactose, amino acids, lipids, and nucleotides. Principal component analysis (PCA) of the samples based on 126 unique compounds demonstrated a clear separation between the *Shp2*^*flox/flox*^ and ^*rod*^*Shp2*^*−/−*^ mice (**D**).

We examined the levels of proteins involved in glucose metabolism in the retinas of *Shp2* ^*flox/flox*^ and ^*rod*^*Shp2*^*−/−*^ mice. The proteins studied included glucose transporter 1 (Glut1), hexokinase 1 (HK-1), hexokinase 2 (HK-2), pyruvate kinase M1 (PKM1), pyruvate kinase M2 (PKM2), Aldolase C (ALDOC), pyruvate dehydrogenase (PDH), lactate dehydrogenase A (LDHA), and lactate dehydrogenase B (LDHB). Immunoblot analysis showed significantly decreased levels of Glut1 and significantly increased levels of HK-2, ALDOC, and PDH in ^*rod*^*Shp2*^*−/−*^ retinas compared to *Shp2* ^*flox/flox*^ retinas (Fig. 6A, B). Additionally, LDHA levels were decreased in ^*rod*^*Shp2*^*−/−*^ retinas compared to *Shp2* ^*flox/flox*^ retinas, though this difference was only marginally significant (*p* < 0.06). We also examined the phosphorylation status of PKM2, LDHA, and PDH, finding no significant differences between ^*rod*^*Shp2*^*−/−*^ and *Shp2* ^*flox/flox*^ mice. Furthermore, we observed significantly increased levels of the Müller cell marker, glial fibrillary acidic protein (GFAP), in ^*rod*^*Shp2*^*-/*^ retinas compared to *Shp2* ^*flox/flox*^ retinas (Fig. 6A, B). Increased GFAP expression has been observed in inflammation [17–19] and thus, we examined the expression of inflammatory markers (*Arg1, Cd206, Cd32, Cd68, Gfap, Il-8, Il-10, Il-1β, Ifn-γ, Inos, Mcp1, Nfh, Tnf-α*) by RT-PCR on RNA prepared from *Shp2* ^*flox/flox*^ and ^*rod*^*Shp2*^*−/−*^ mouse retinas using primers listed in Table 1. Our results indicate significantly increased gene expression of *Gfap* consistent with the immunoblot, *Il-8* and *Il-10*, and *Il-1β* in ^*rod*^*Shp2*^*−/−*^ mouse retina compared to *Shp2* ^*flox/flox*^ mouse retina (Fig. 6C).

**Fig. 7:**
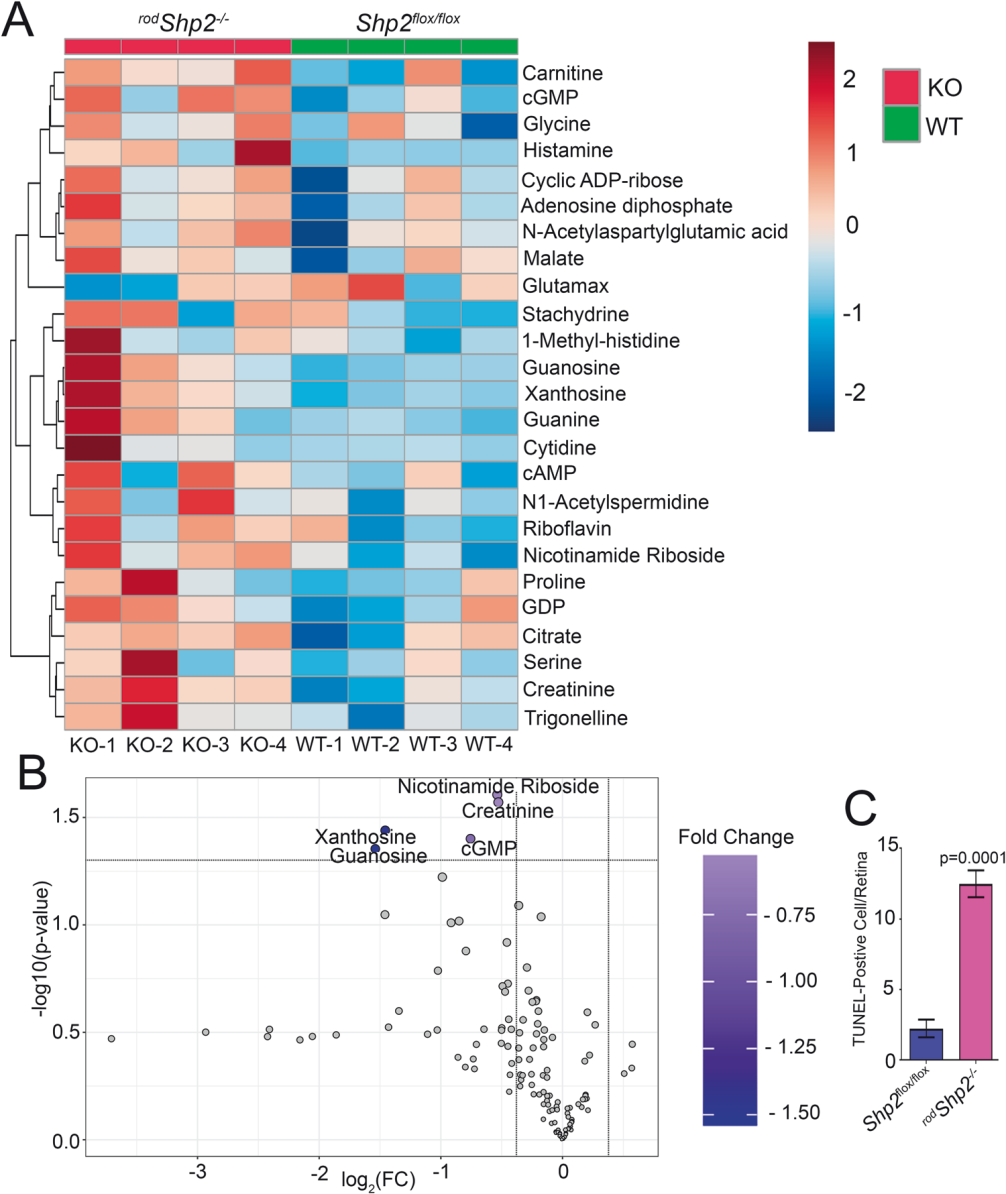
Changes in steady-state level retinal metabolites in mouse retina lacking Shp2. Steady-state levels of retinal metabolites were measured by LC/MS from four-independent *Shp2*^*flox/flox*^ (WT) and ^*rod*^*Shp2*^*−/−*^ (KO) mouse retinas. A heatmap of changed metabolites in ^*rod*^*Shp2*^*−/−*^ mouse retina (**A**). Volcano plot showing differential metabolites in ^*rod*^*Shp2*^*−/−*^compared to *Shp2*^*flox/flox*^ mouse retina, horizontal line indicates changes with (*p* < 0.05) (**B**). Quantification of TUNEL staining in ^*rod*^*Shp2*^*−/−*^mouse retinas. Prefer-fixed retinal sections of *Shp2*^*flox/flox*^ and ^*rod*^*Shp2*^*−/−*^ mice were examined for cell death with in-situ localization of apoptosis using TUNEL and counted the number of TUNEL-positive cells over the entire retina (**C**). Data are mean ± *SEM*, (*n* = *4*).

To determine the effect of loss of Shp2 in rods on retinal metabolism, we measured steady-state levels of metabolites in 6-week-old *Shp2* ^*flox/flox*^ and ^*rod*^*Shp2*^−/−^ mouse retinas. We have employed LC-MS to examine steady-state levels of 126 metabolites that are representative of major pathways in metabolism, including glucose, lactose, amino acids, lipids, and nucleotides. Principal component analysis (PCA) of the samples based on 126 unique compounds demonstrated a clear separation between the *Shp2* ^*flox/flox*^ and ^*rod*^*Shp2*^−/−^ groups (Fig. 6D). Our analysis indicated that several metabolites were increased in ^*rod*^*Shp2*^−/−^ mouse retinas compared to Shp2 ^*flox/flox*^ mouse retinas (Fig. 7A). Of all the metabolites, Xanthosine, Guanosine, Nicotinamide Riboside, Creatinine and cGMP levels were significantly higher in ^*rod*^*Shp2*^−/−^ mouse retina compared to *Shp2* ^*flox/flox*^ mouse retina (Fig. 7B). Since increased cGMP levels can cause photoreceptor degeneration [10, 20], we investigated whether Shp2 loss leads to cell death in rod photoreceptor cells using TUNEL labeling. Our results showed significantly increased cell death in ^*rod*^*Shp2*^−/−^ mice compared to *Shp2* ^*flox/flox*^ mice (Fig. 7C). These findings suggest that Shp2 is essential for rod photoreceptor survival.

## Discussion

In our current study, we meticulously characterized the cell-specific expression of Shp2 using an unbiased TRAP technique. We identified the highest enrichment of Shp2 in photoreceptor cells compared to many other retinal cell types, including Müller cells. Additionally, staining of isolated bovine rod photoreceptors for Shp2 confirmed this observation. Shp2 is crucial for early optic cup patterning at embryonic day 9.0 (E9.0) [21]. However, Six3-Cre-mediated ablation of Shp2 after E10.5 does not affect retinal development [21]. Despite this, *Six3-Cre; Shp2*^*fox/flox*^ mutants exhibit ocular defects at weaning (P21), including optic nerve thinning, a dystrophic optic stalk, vacuoles, reduced retinal thickness, and rosettes in the outer nuclear layer [7]. Increased apoptosis occurs in various retinal layers from P10 to P21 [7]. Shp2 knockout mice generated in this study under the control of the rhodopsin promoter, which is expressed postnatally at day 5 [22], did not exhibit retinal abnormalities or rosette formation at 6 weeks. However, by 20 weeks, they exhibited age-related photoreceptor degeneration and functional loss, indicating Shp2’s cell-autonomous role in rod photoreceptors.

The tight-junction protein OCLN, identified as a substrate of Shp2 [11], is known to be expressed in the RPE [23] and observed in the outer limiting membrane [24] within the retina. Our biochemical characterization and TRAP analysis showed that OCLN expression is present in RPE, rods, and cones, but not in Müller cells. We observed a reduction in OCLN expression in the retina of ^*rod*^*Shp2*^−/−^ mouse, and there was also a loss of this protein in the RPE. It has been shown that OCLN phosphorylation regulates its assembly into the tight junctions [25]. In the intact epithelium, OCLN exhibits high phosphorylation on serine and threonine residues, while tyrosine phosphorylation is minimal [25]. However, when tight junctions are disrupted, OCLN serine/threonine residues undergo dephosphorylation, and there is an increase in tyrosine phosphorylation in OCLN [25]. In line with previous research, an in vitro study showed that when OCLN is phosphorylated at tyrosine, it no longer interacts with ZO-1, another junctional protein [25]. These findings suggest that the equilibrium between tyrosine kinases and phosphatases plays a crucial role in determining OCLN phosphorylation and the function of tight junctions. Given that Shp2 is a tyrosine phosphatase, the observed reduction of OCLN in the retina and RPE of ^*rod*^*Shp2*^−/−^ mice may indicate that OCLN could persist in a state of tyrosine phosphorylation, potentially causing an increased turnover. Additional comprehensive studies are necessary to confirm this hypothesis.

The TRAP/RNA sequencing highlights that Shp2 regulates several important signaling pathways in rod photoreceptor cells. The pathway analysis also identified the likely activation of upstream regulators, MLX interacting protein-like (Mlxipl), and serine/threonine kinase 11 (Stk11), and the likely inhibition of upstream regulators COP9 Signalosome Subunit 5 (Cops5), and protein tyrosine phosphatase receptor type R (Ptprr). Our study indicates that the majority of pathways regulated by Shp2 are associated with the Cell Cycle. Previous studies have identified a novel connection between Shp2 and cell cycle checkpoints [26]. Additionally, Shp2 has been found to enhance the proliferation of breast cancer cells by regulating Cyclin D1 stability through the PI3K/Akt and GSK3β pathway [27]. Moreover, the G2/M checkpoint induced by DNA damage in SV40 large T antigen-immortalized embryonic fibroblast cells is dependent on Shp2 [28]. It is noteworthy that photoreceptor cells are postmitotic, and further investigations are required to comprehend the involvement of Shp2-regulated cell cycle regulatory pathways in photoreceptor functions.

Carbohydrate-responsive element-binding protein (ChREBP), also known as MLX-interacting protein-like (MLXIPL), is a key transcriptional regulator [29] activated by glucose, independent of insulin [30]. In adipose tissue, ChREBP promotes de novo lipogenesis from glucose [31], while in the liver, it induces glycolysis and lipogenesis [30]. Our study indicates that the loss of Shp2 in rod cells impacts glycolysis, the pentose phosphate pathway, the TCA cycle, and mitochondrial metabolism. In line with these findings, we also noted changes in the levels of the Glut1, glucose sensor ALDOC, hexokinase 2, and PDH in the retinas of ^*rod*^*Shp2*^−/−^ mice.

In ^*rod*^*Shp2*^*−/−*^ mice, increased GFAP expression indicates Müller cell activation. This activation, known as gliosis, occurs during ocular inflammation and involves interactions with immune and microglial cells [32]. We also observed elevated inflammatory markers, but our rod photoreceptor-TRAP data showed that these markers (*IL-8, IL-10, and IL-1beta*) were not expressed in photoreceptor cells, suggesting they originate from non-photoreceptor cells, possibly Müller cells. Further studies are needed to understand how Shp2 regulates retinal inflammation.

The retinal degeneration observed in ^*rod*^*Shp2*^*−/−*^ mice may be due to reduced levels of Glut1 and increased levels of cGMP. Glut1 facilitates glucose transport from the RPE to photoreceptor cells [33], and reduced glucose supply contributes to photoreceptor cell death in inherited and age-related retinal diseases [33]. Specifically, the deletion of Glut1 in rod photoreceptors restricts glucose transport to the outer retina, leading to photoreceptor degeneration and impaired outer segment renewal [33]. Further studies are needed to determine if Shp2 dephosphorylates Glut1 to maintain its stability and function. Increased levels of cGMP have been shown to induce photoreceptor degeneration [34] and our metabolic analysis shows increased levels of cGMP in mouse rods lacking Shp2. Further studies are needed to examine how loss of Shp2 increases the levels of cGMP. Our study emphasizes the crucial role of Shp2 in photoreceptor cell functions, with potential phosphatase-independent functions [35] that require further investigation.

## Limitations of this study

We observed decreased Occludin (OCLN) levels in the retina and reduced expression in the RPE, but could not assess the interaction between OCLN and Shp2 on photoreceptor function due to the lack of floxed OCLN mice. Although sex-specific metabolomics differences in mouse eyes have been reported [36], our current metabolomics data is not separated by sex. We plan to address this in future studies with a larger sample size.

## Materials and Methods

### Antibodies

Polyclonal Shp2 (Cat # 20145-1-AP), LDHA (Cat # 21799-1-AP), LDHB (Cat # 14824-1-AP), and occludin (Cat # 27260-1-AP) antibodies were purchased from Proteintech (Rosemont, IL). Polyclonal hexokinase 1 (Cat # 2024), hexokinase 2 (Cat # 2867), phospho-PKM2 (Y105) (Cat # 3827), PKM2 (Cat # 4053), PKM1 (Cat # 7067), phospho-LDHA (Tyr10) (Cat # 8176), PDH (Cat # 3205) and phospho-PDH (Ser293) (Cat # 31866) antibodies were obtained from Cell Signaling (Danvers, MA). Monoclonal actin antibody (Cat # MA1-744) was purchased from Thermo Fisher Scientific (Dallas, TX). Polyclonal Glut1 antibody (Cat # NB-110-39113) was procured from Novus Biologicals (Littleton, CO). Polyclonal glial fibrillary acid protein (GFAP) antibody (Cat # 20334) was purchased from Dako (Santa Clara, CA). Rabbit polyclonal Cre antibody (Cat # 69050-3) suitable for Western blot analysis was purchased from Novagen (Darmstadt, Germany). Anti-rabbit and mouse secondary antibodies were obtained from Millipore (Billerica, MA). Monoclonal 1D4 rhodopsin antibody was a kind gift from Dr. James F. McGinnis (University of Oklahoma Health Sciences Center). Polyclonal anti-Rpe65 antibody was a kind gift from Dr. Jing-Xing Ma (University of Oklahoma Health Sciences Center). DAPI used for nuclear staining was procured from Invitrogen-Molecular Probes (Carlsbad, CA).

### Animals

All animals were treated following the *ARVO Statement for using Animals in Ophthalmic and Vision Research* and the *NIH Guide for the Care and Use of Laboratory Animals*. The protocols were approved by the IACUC at the University of Oklahoma Health Sciences Center. Breeding pairs of *PTPN11* (Jax # 025758), RiboTag (Jax #011029), and tamoxifen-inducible RaxCre^ER^ mice (Jax # 025521) were purchased from The Jackson Laboratory (Bar Harbor, Maine). The rhodopsin-Cre (i75Cre) mice have been described earlier [37] and were provided by Dr. Ching-Kang Jason Chen (Baylor College of Medicine, Houston, TX). Tetracycline-inducible VMD2-Cre mice and human red/green cone opsin promoter-Cre mice were kindly provided by Dr. Yun Le (OUHSC). Animals were born and raised in our vivarium and kept under dim cyclic light (40-60 lux, 12 h light/dark cycle). All mice were screened for *rd1* and *rd8* mutations and were negative for these mutations. The mice were deeply anesthetized, and the retinas were harvested. The mice were euthanized by CO_2_ asphyxiation. The retinas were used for RNA and protein isolation, and enucleated eyes were used for immunohistochemistry. To eliminate bias, we randomly assigned mice of the same sex, age, and genetic strain to each experimental group. We mixed litters to prevent litter bias. After genotyping and assigning unique eartag identifiers, Dr. Rajala, the principal investigator, randomly selected the cohorts. This ensured that the research personnel conducting the experiments were blinded and only knew the mice by their eartag numbers.

### Generation of conditional knockout and retinal cell-specific RiboTag mice

We have generated rod photoreceptor-specific *PTPN11* (Shp2) knockout mice by breeding floxed Shp2 mice with mice carrying Cre-recombinase under the control of a rhodopsin promoter. For the identification of genotypes, we used the following primers. Rhodopsin-Cre (forward primer: TCAGTGCCTGGAGTTGCGCTGTGG and reverse primer: CTTAAAGGCCAGGGCCTGCTTGGC) and Shp2 floxed (forward primer: ATGACTCCTGAAGCCCATTG and reverse primer CTTCCCATCACCTCAGCATCC). The rhodopsin-Cre will identify 500 bp product. The Shp2 genotyping will generate mutant 320 bp; heterozygous 221 and 320 bp and wild type 221 bp, respectively. The RiboTag mouse carries a ribosomal protein gene (*Rpl22*) with a floxed C-terminal exon followed by an identical exon tagged with hemagglutinin (HA) epitope [38]. We bred RiboTag mice with mice carrying Cre-recombinase under the control of rod (rhodopsin), cone (red/green opsin), RPE (VMD2), Müller cells (Rax), and ganglion cell (CMV packaged into an AAV vector, Vector Biolabs)-specific promoters. The isolation of polyribosomes containing actively translating mRNAs and qRT-PCR analysis was carried out as described [9].

### Statistical Analysis

We determined the sample size through power analysis [39]. Data underwent comprehensive statistical evaluation using GraphPad Prism 7.3 software. No samples were excluded from the analysis. Various statistical methods were employed based on the experiment type. Before analysis, we conducted normality tests (Anderson-Darling, D’Agostino & Pearson, Shapiro-Wilk, Kolmogorov-Smirnov) to assess data distribution (normal or non-normal). If non-normally distributed, an unpaired non-parametric test was applied for group comparisons, involving multiple Mann-Whitney U tests. To address multiple comparisons, a false discovery rate was controlled at Q = 1%, implementing the two-stage Benjamini-Krieger-Yekutieli method. For normally distributed data, parametric tests were conducted using Welch’s correction to assess significance between the two groups. The resulting p-values were used for inference. One-way ANOVA determined statistical differences among means of three or more independent groups, while a two-way ANOVA assessed how a quantitative variable’s mean changes with two categorical variables.

### Other methods

We conducted Translation Ribosomal Affinity Purification, electroretinography (ERG), histological analyses, immunoblotting, and immunohistochemistry, following the procedures described earlier [9, 22, 40]. Targeted metabolomics was carried out by LC-MS as described [36]. We added Nicotinamide-D4 as the internal standard for quality control.

### Supplementary information

Uncropped images

## Data Availability

All data generated or analyzed during this study are included in this published article. The TRAP data reported here have been deposited in the Gene Expression Omnibus with accession number GSE269514. The metabolomics LC/MS Mass Spectrometry data has been deposited in the MassIVE Repository under Reference number MSV000094985.
