# Supplementary material for: Age-related retinal degeneration resulting from the deletion of Shp2 tyrosine phosphatase in photoreceptor neurons
Source: Cell Death Dis. 2024 Aug 8;15(8):577. doi: 10.1038/s41419-024-06924-y (PMC11310310; doi:10.1038/s41419-024-06924-y)
Supplement: Supplementary file 1 — Uncropped images [file 41419_2024_6924_MOESM1_ESM.pptx]

## Slide 1
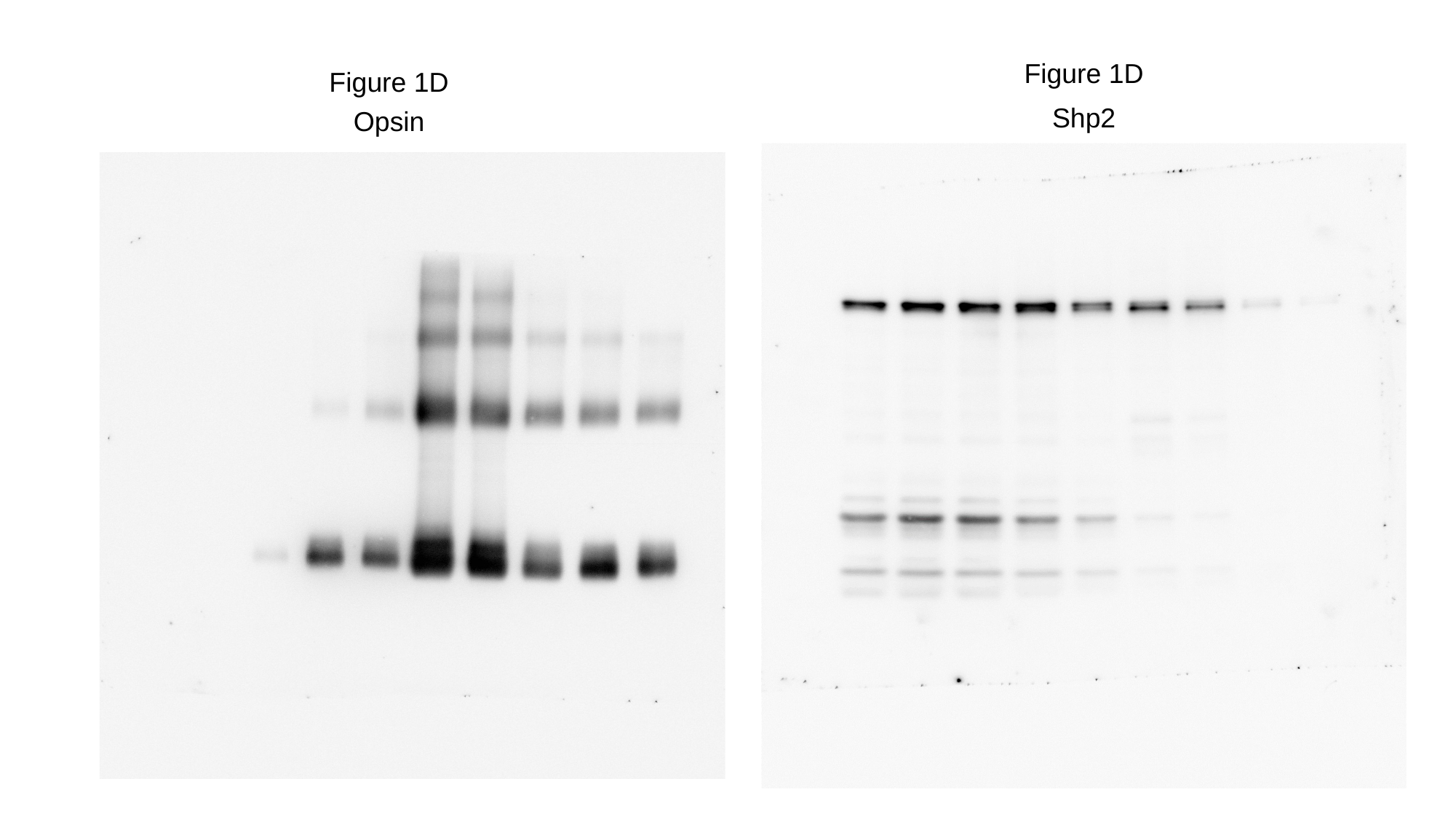

Figure 1D
Figure 1D
Shp2
Opsin

## Slide 2
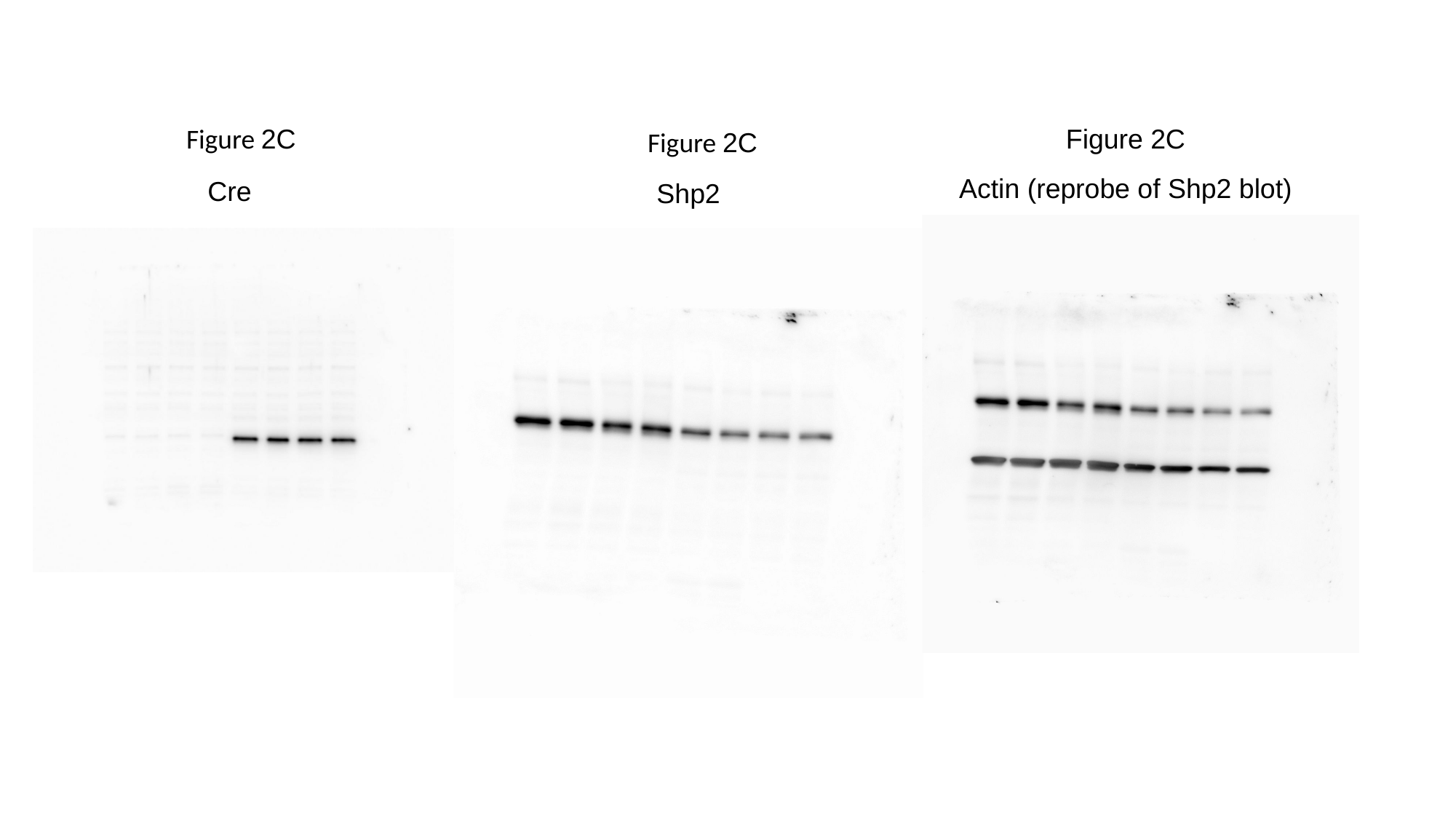

Figure 2C
Figure 2C
Figure 2C
Actin (reprobe of Shp2 blot)
Cre
Shp2

## Slide 3
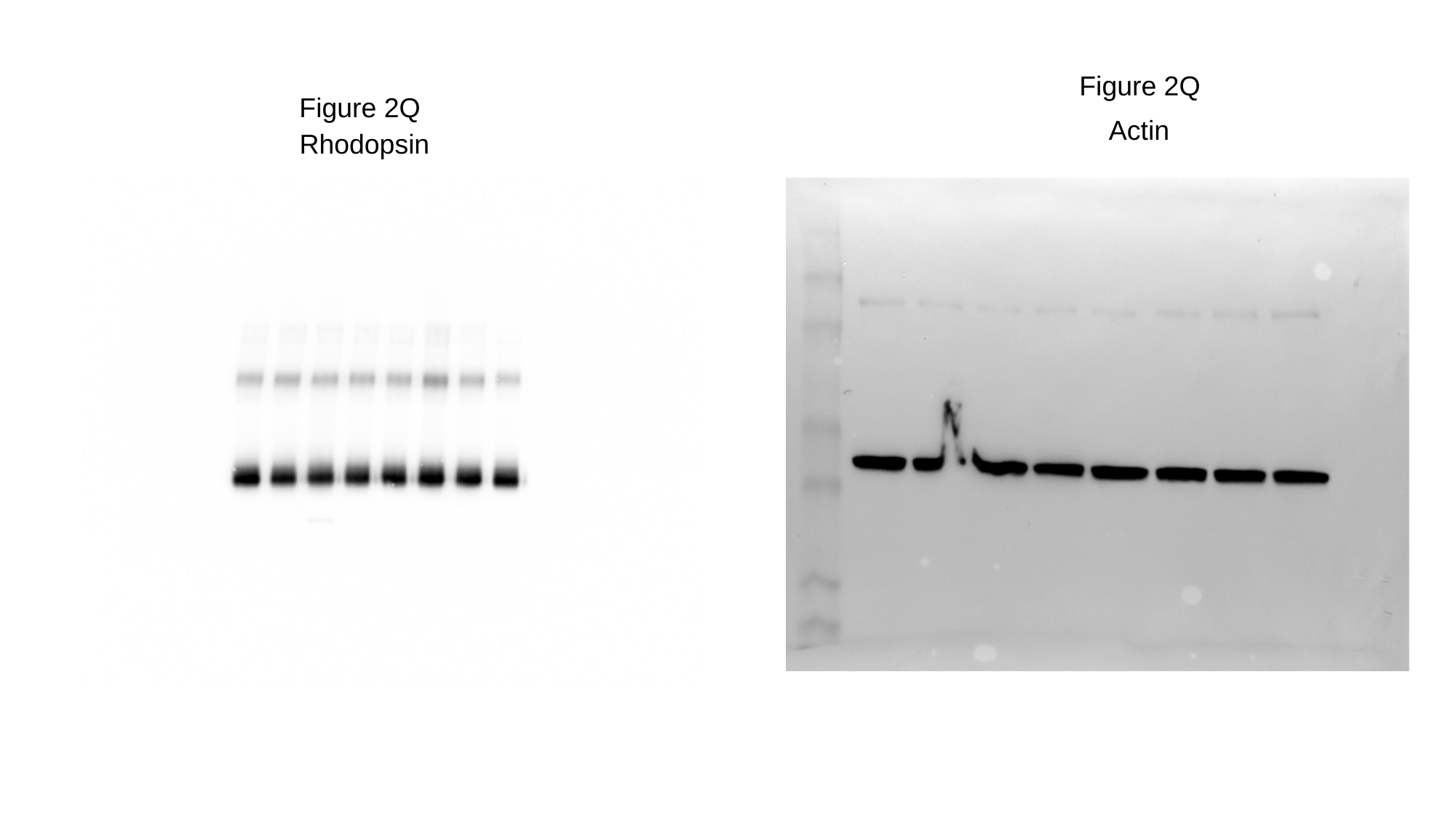

Figure 2Q
Figure 2Q
Actin
Rhodopsin

## Slide 4
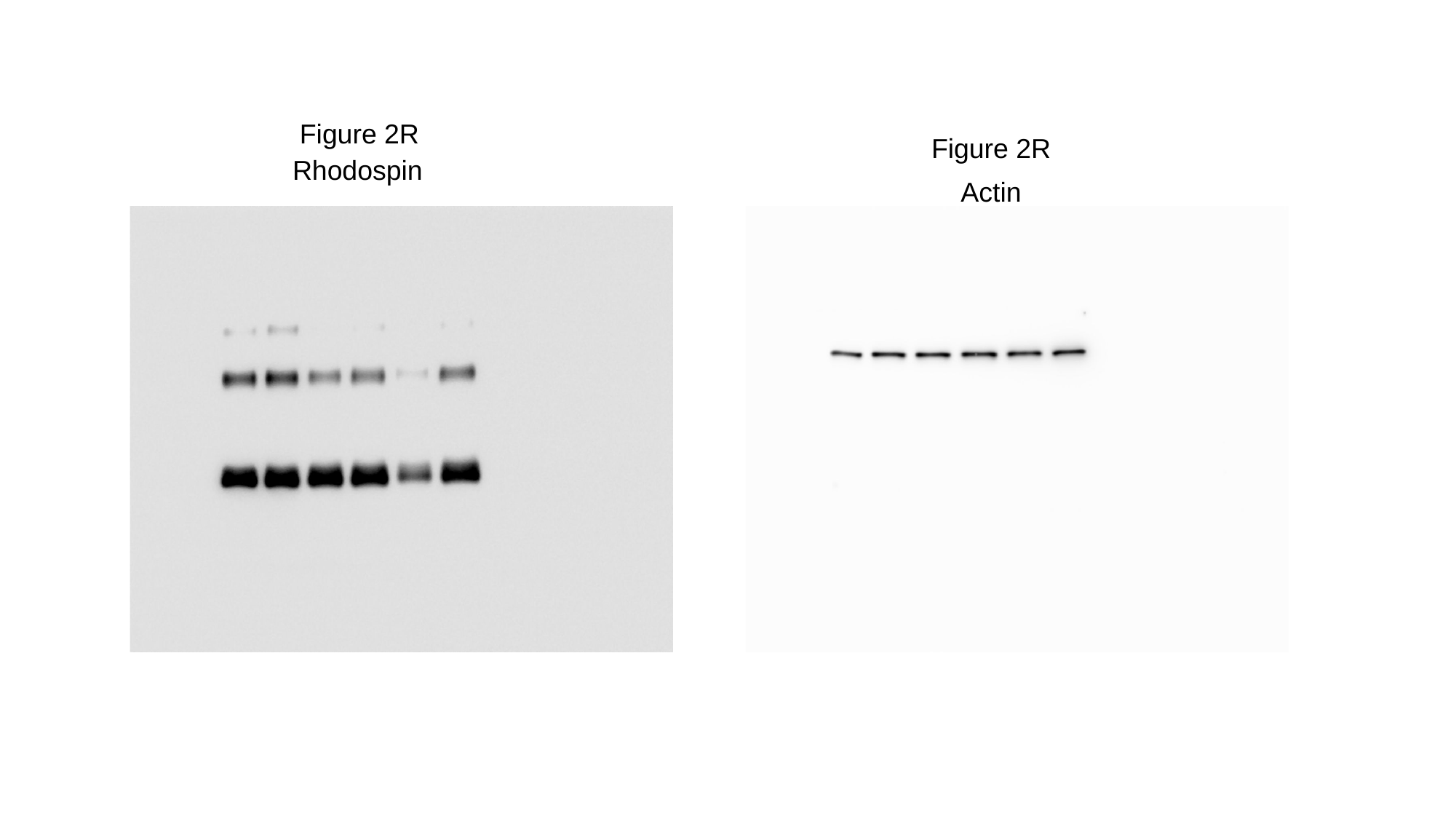

Figure 2R
Figure 2R
Rhodospin
Actin

## Slide 5
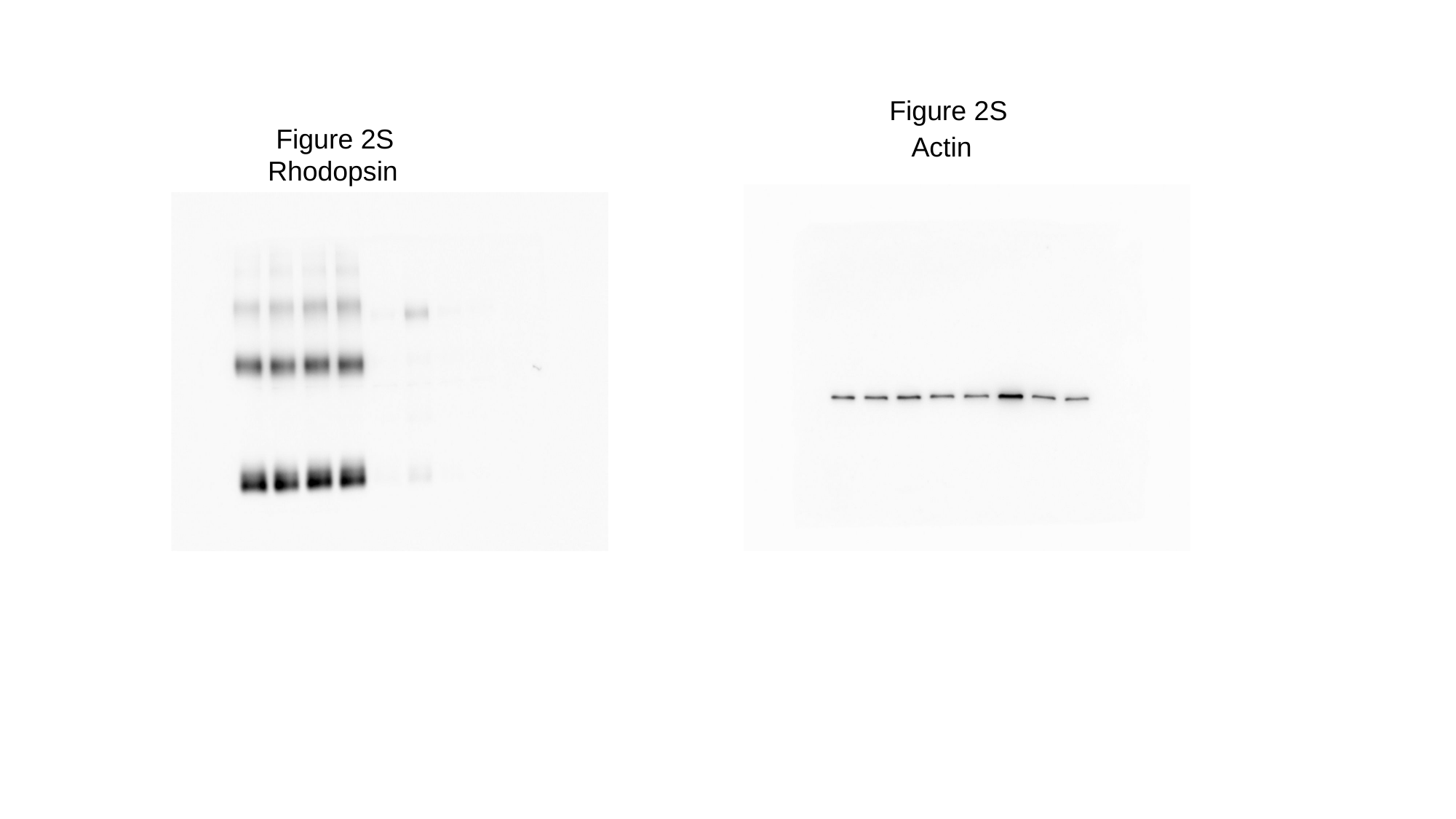

Figure 2S
Figure 2S
Actin
Rhodopsin

## Slide 6
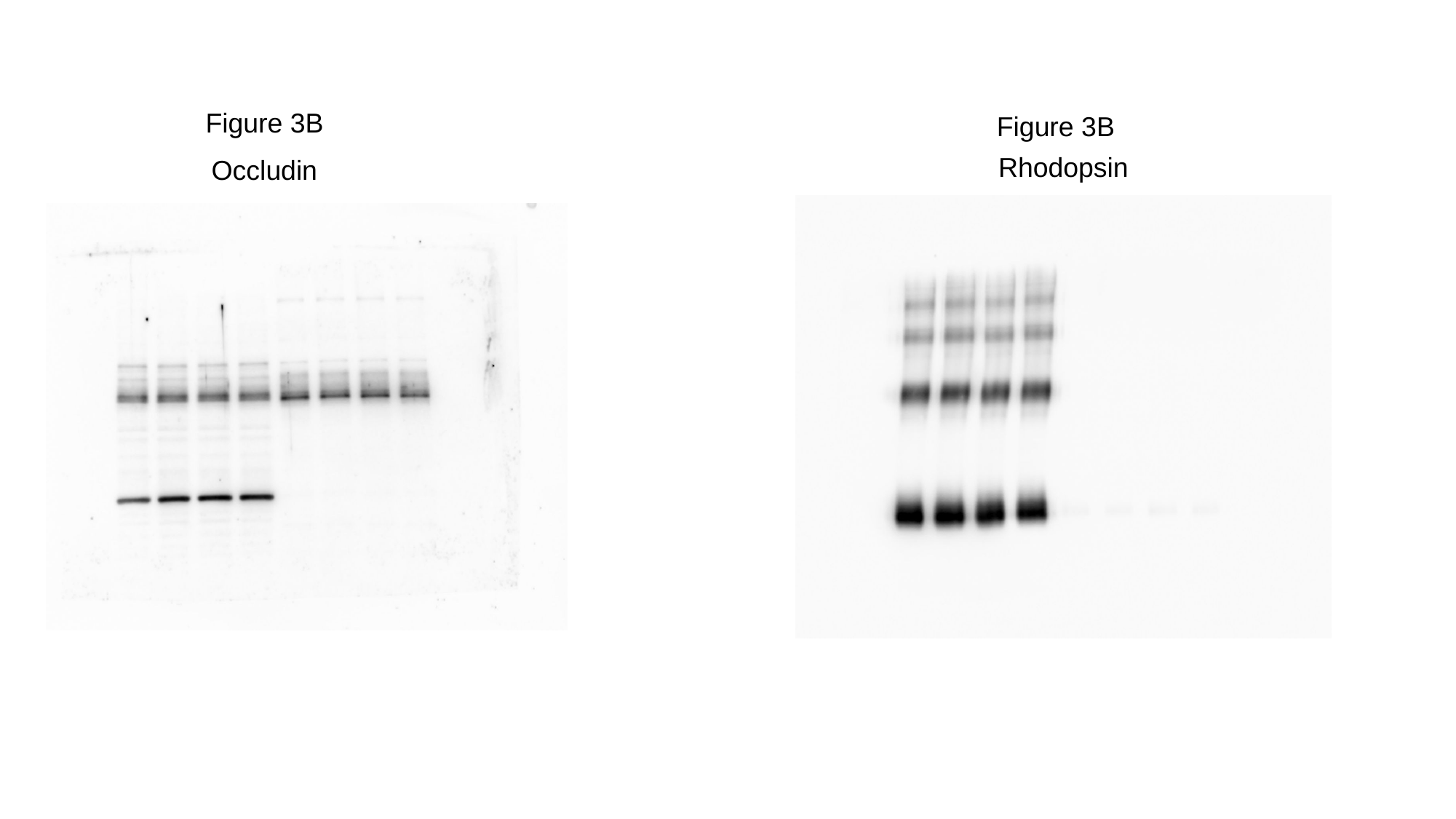

Figure 3B
Figure 3B
Rhodopsin
Occludin

## Slide 7
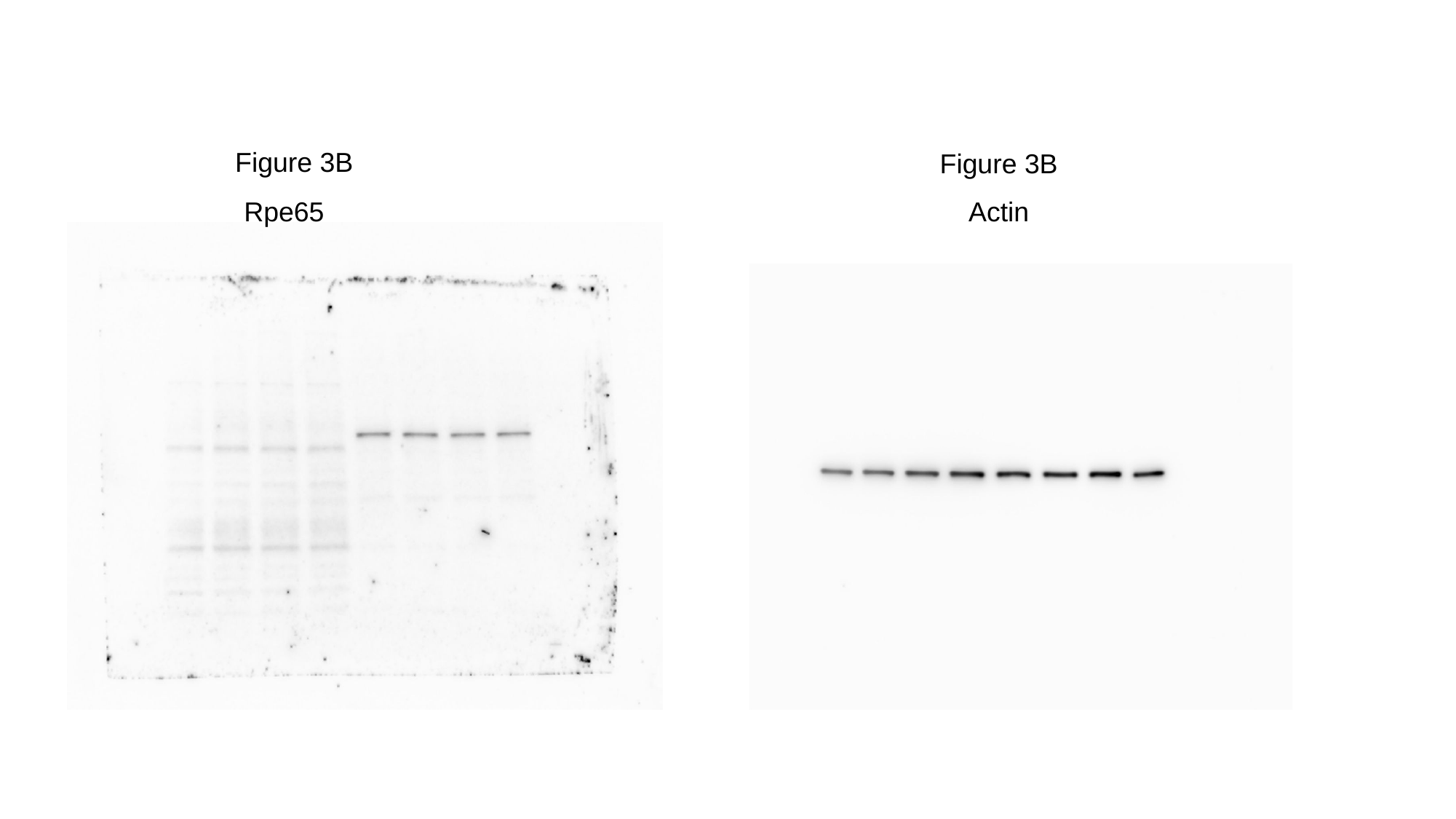

Figure 3B
Figure 3B
Rpe65
Actin

## Slide 8
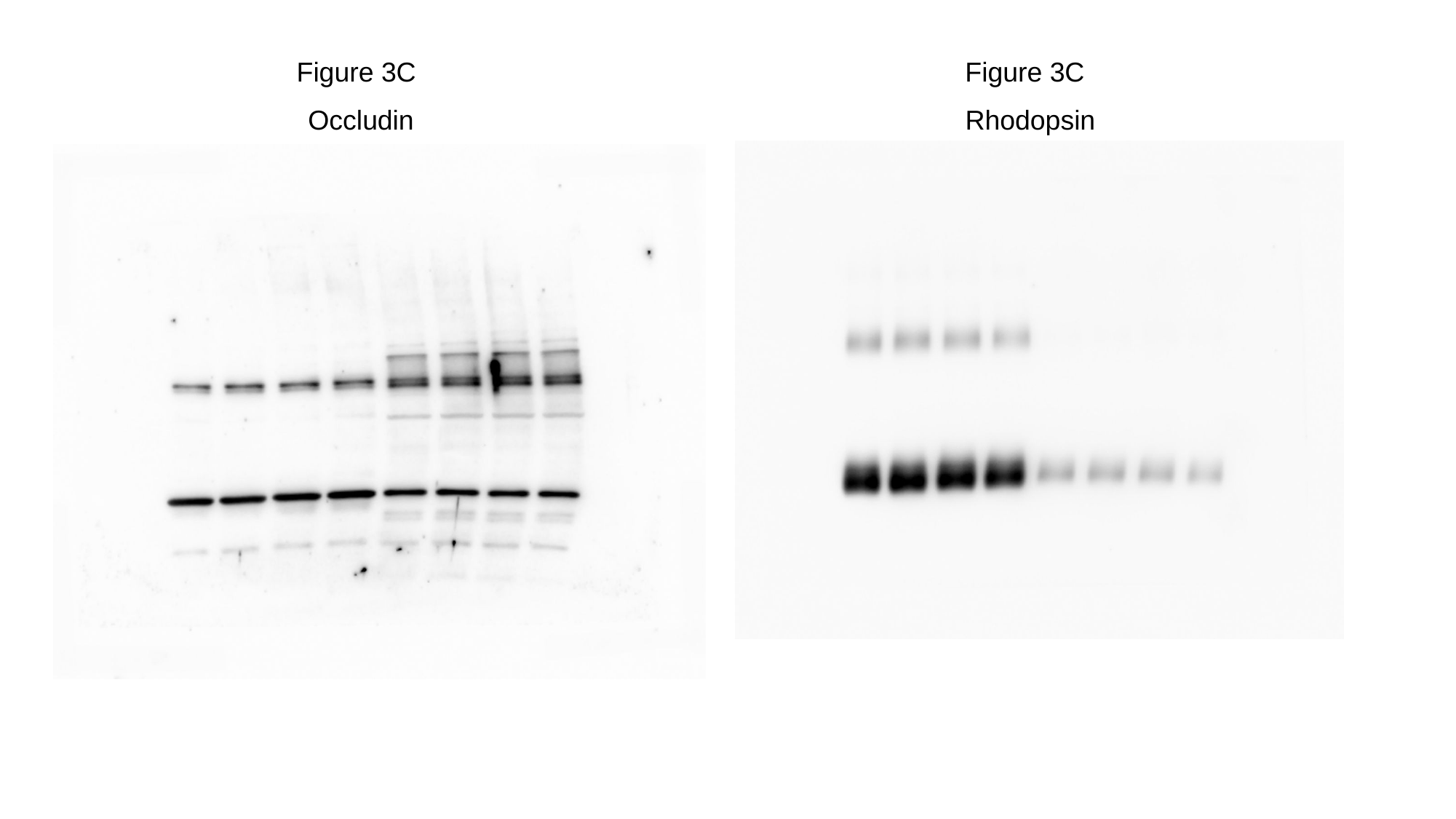

Figure 3C
Figure 3C
Occludin
Rhodopsin

## Slide 9
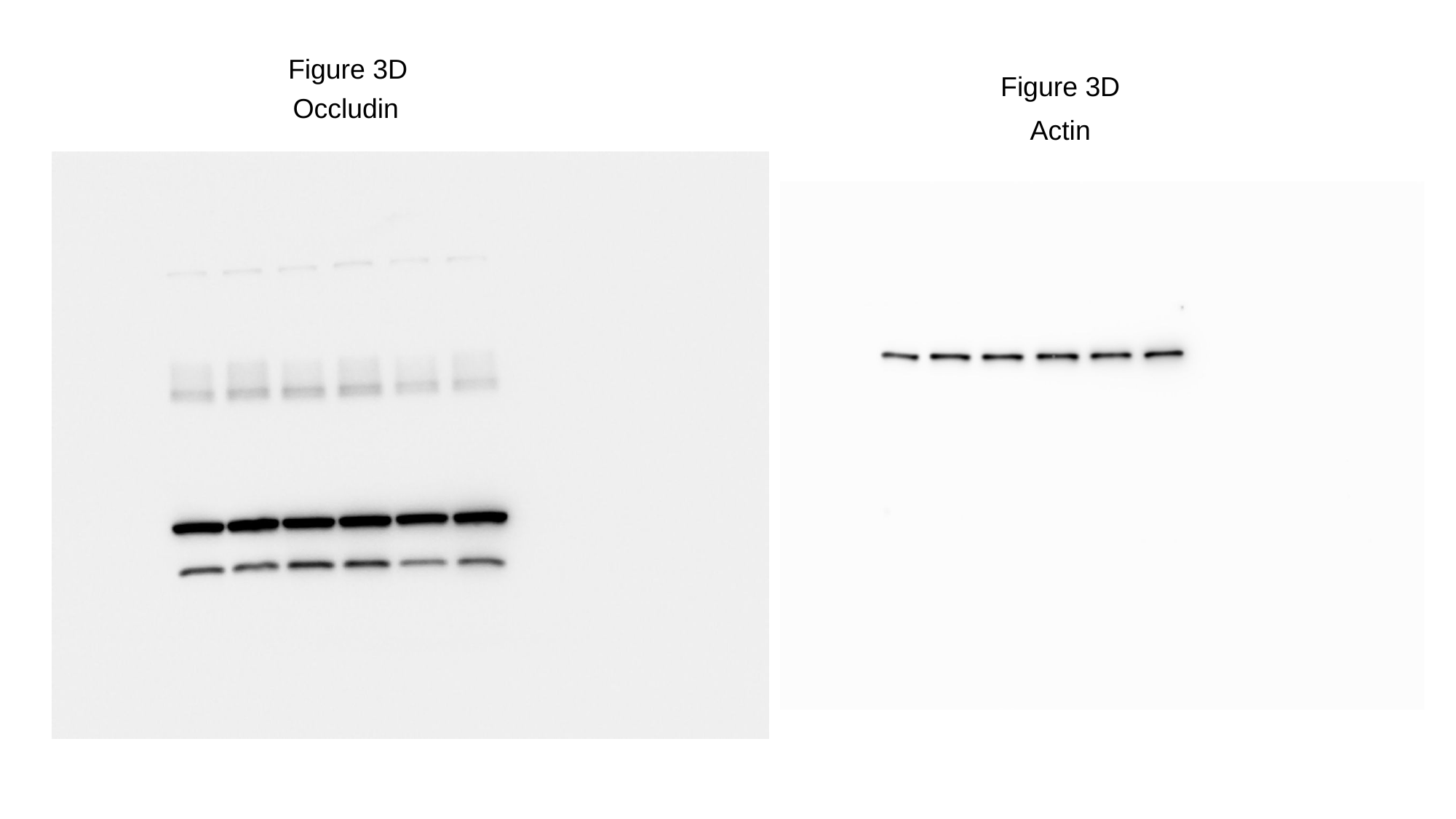

Figure 3D
Figure 3D
Occludin
Actin

## Slide 10
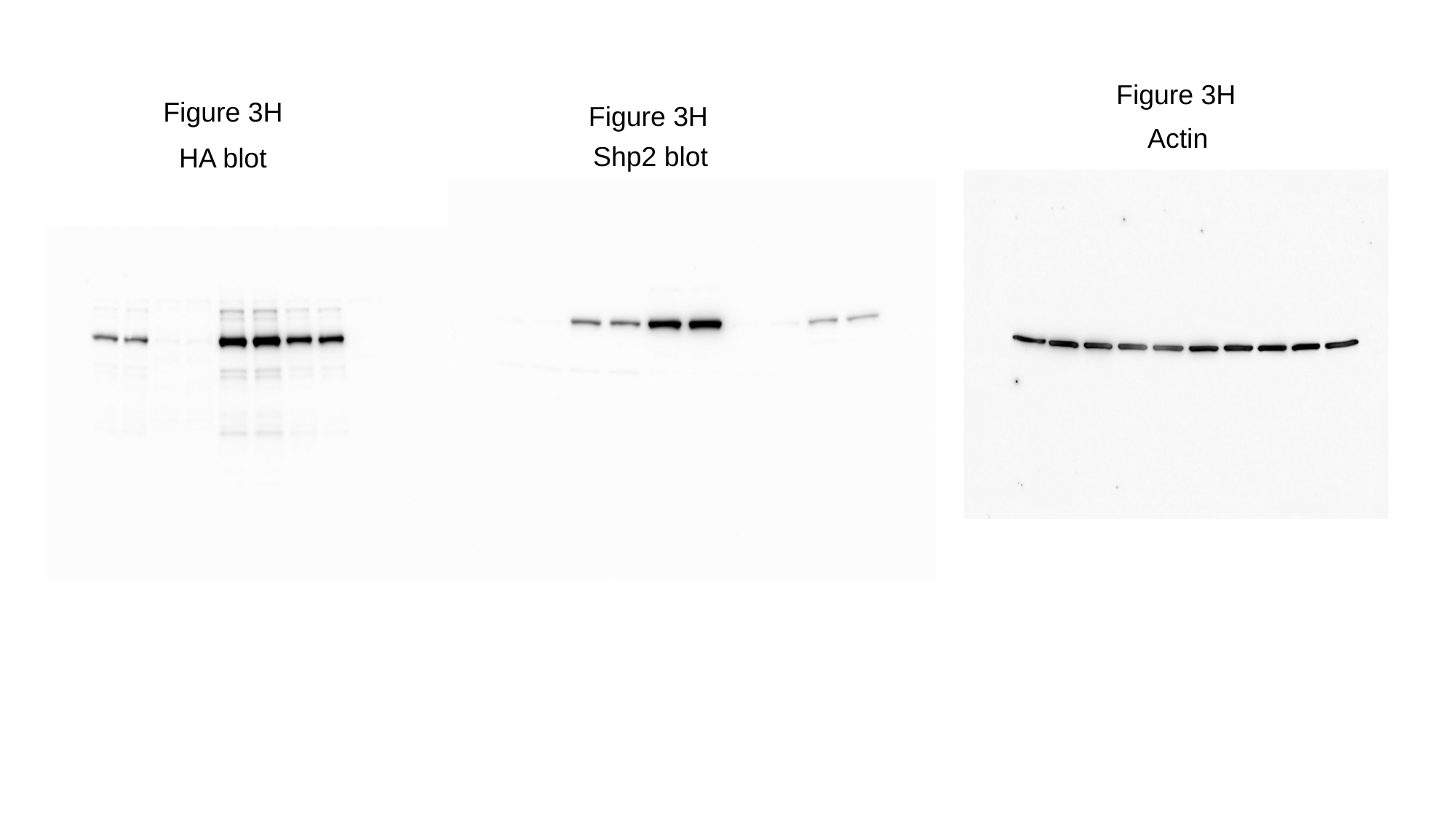

Figure 3H
Figure 3H
Figure 3H
Actin
Shp2 blot
HA blot

## Slide 11
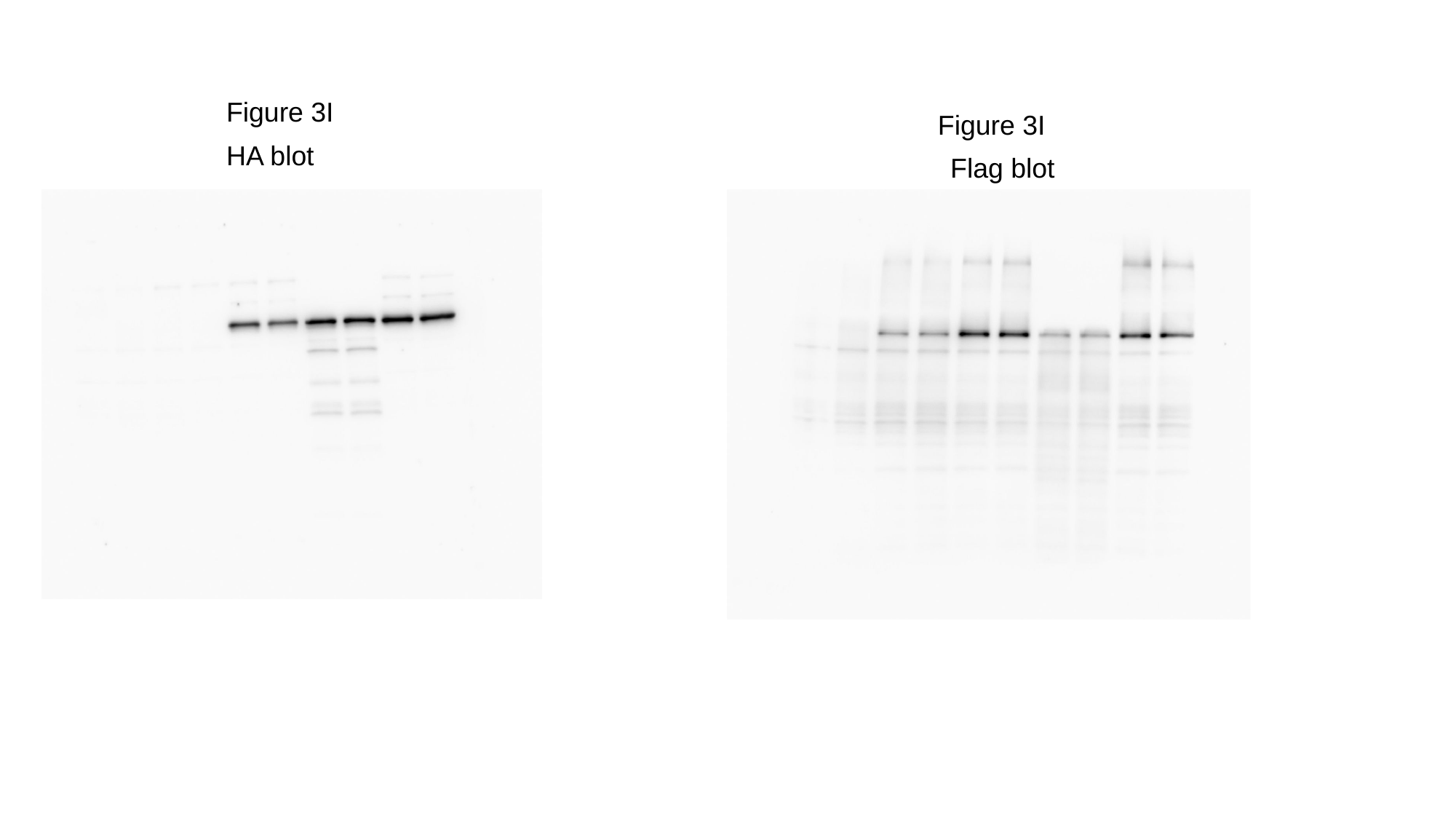

Figure 3I
Figure 3I
HA blot
Flag blot

## Slide 12
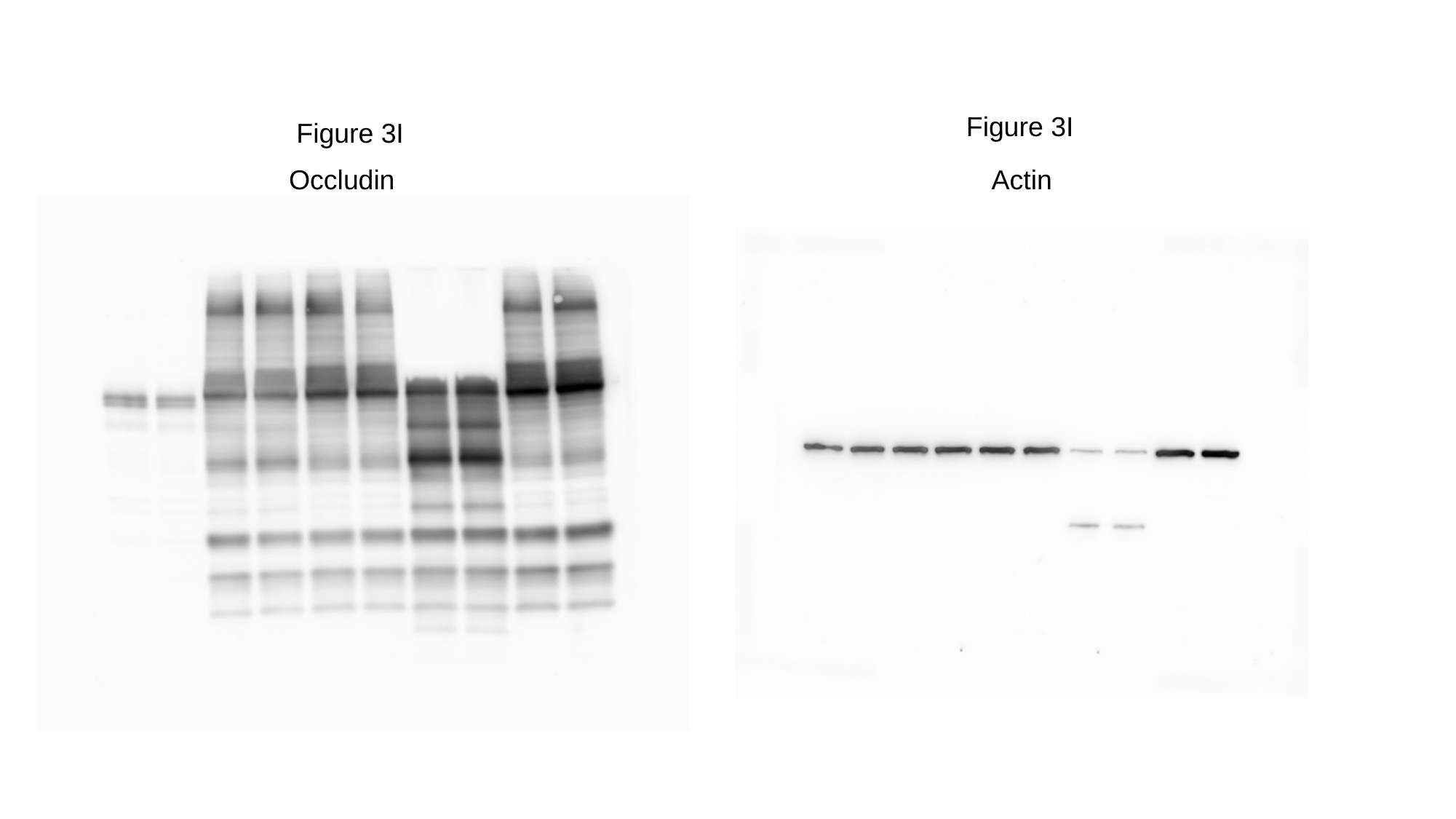

Figure 3I
Figure 3I
Occludin
Actin

## Slide 13
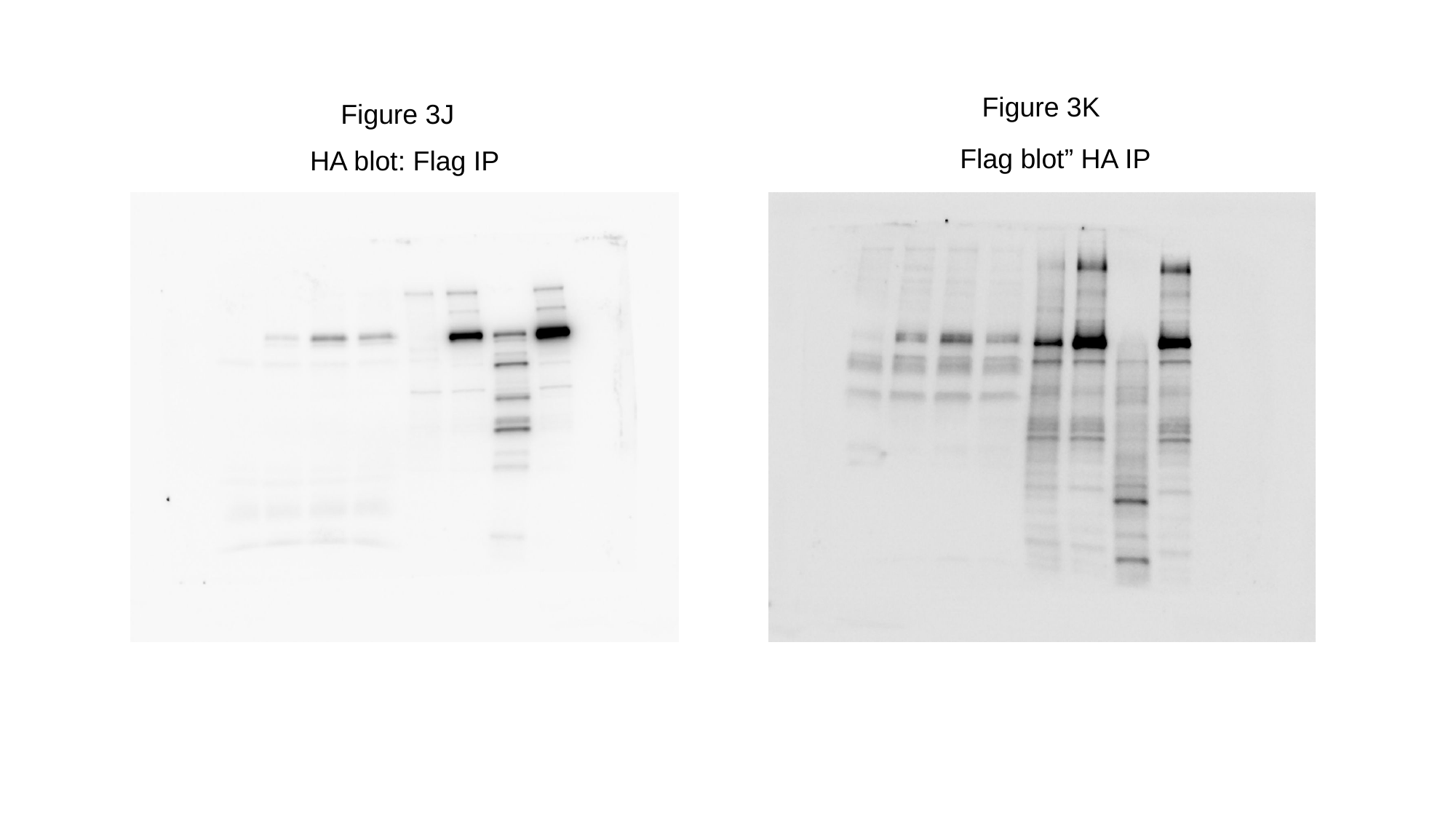

Figure 3K
Figure 3J
Flag blot” HA IP
HA blot: Flag IP

## Slide 14
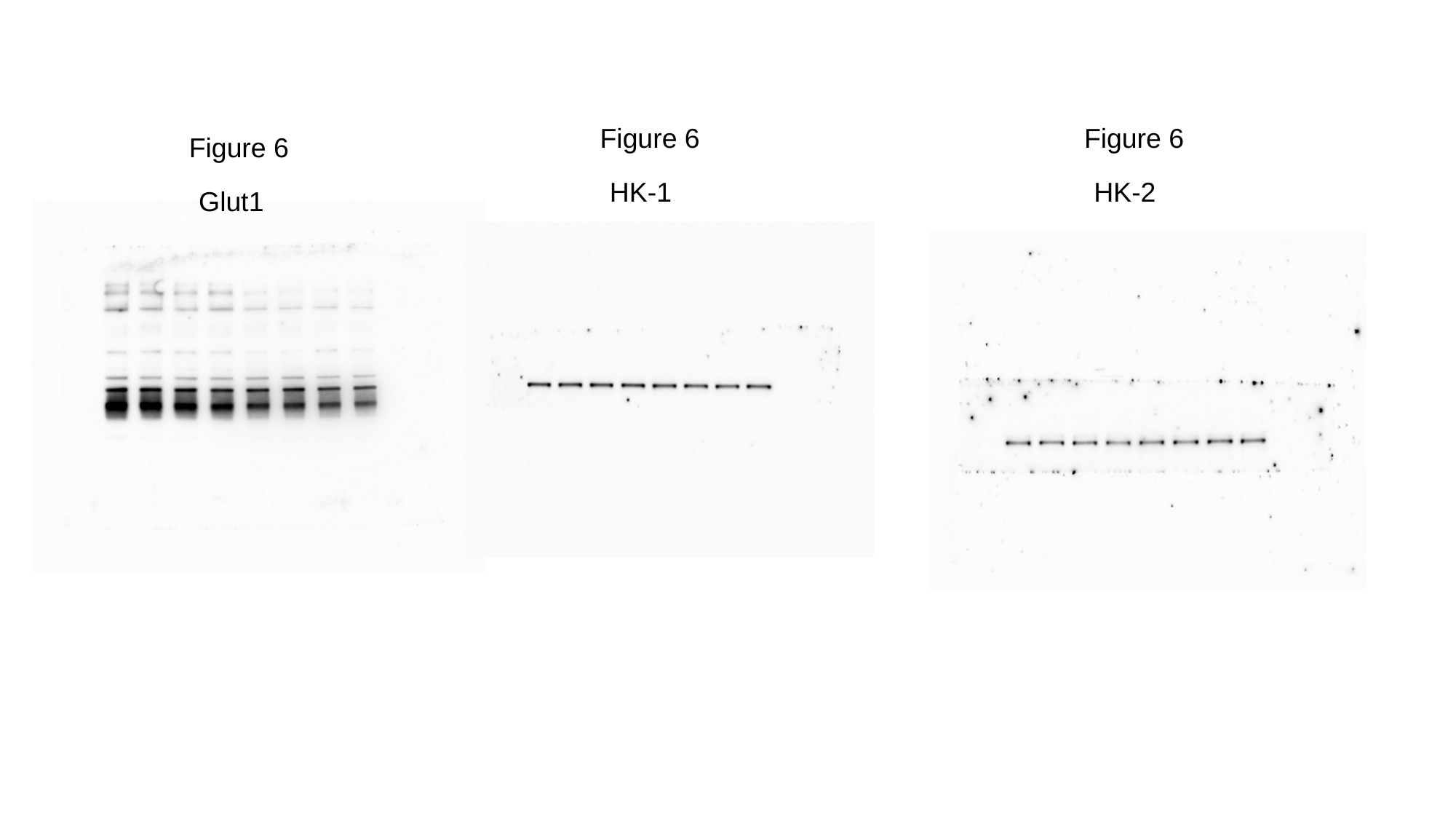

Figure 6
Figure 6
Figure 6
HK-1
HK-2
Glut1

## Slide 15
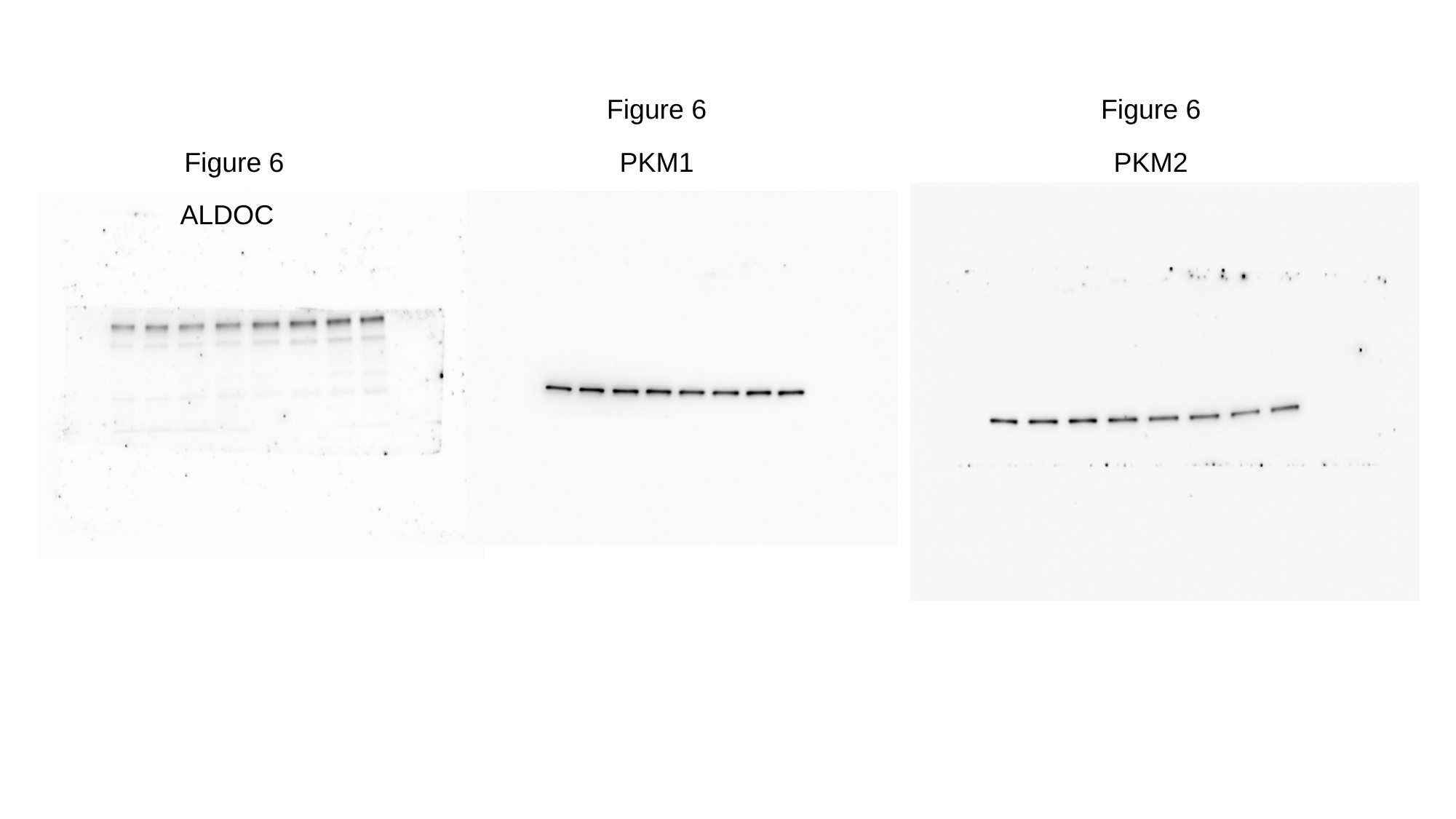

Figure 6
Figure 6
Figure 6
PKM1
PKM2
ALDOC

## Slide 16
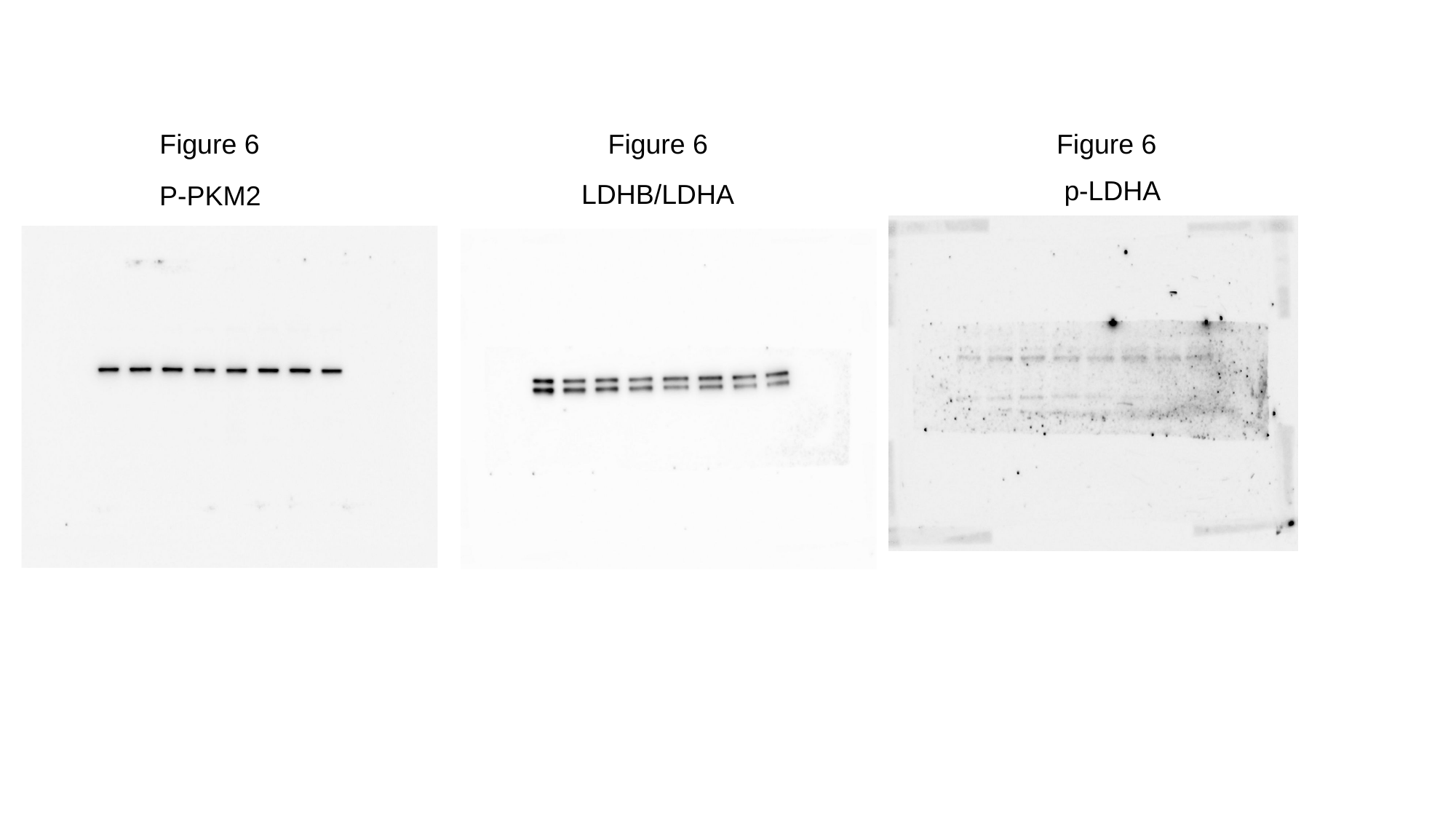

Figure 6
Figure 6
Figure 6
p-LDHA
LDHB/LDHA
P-PKM2

## Slide 17
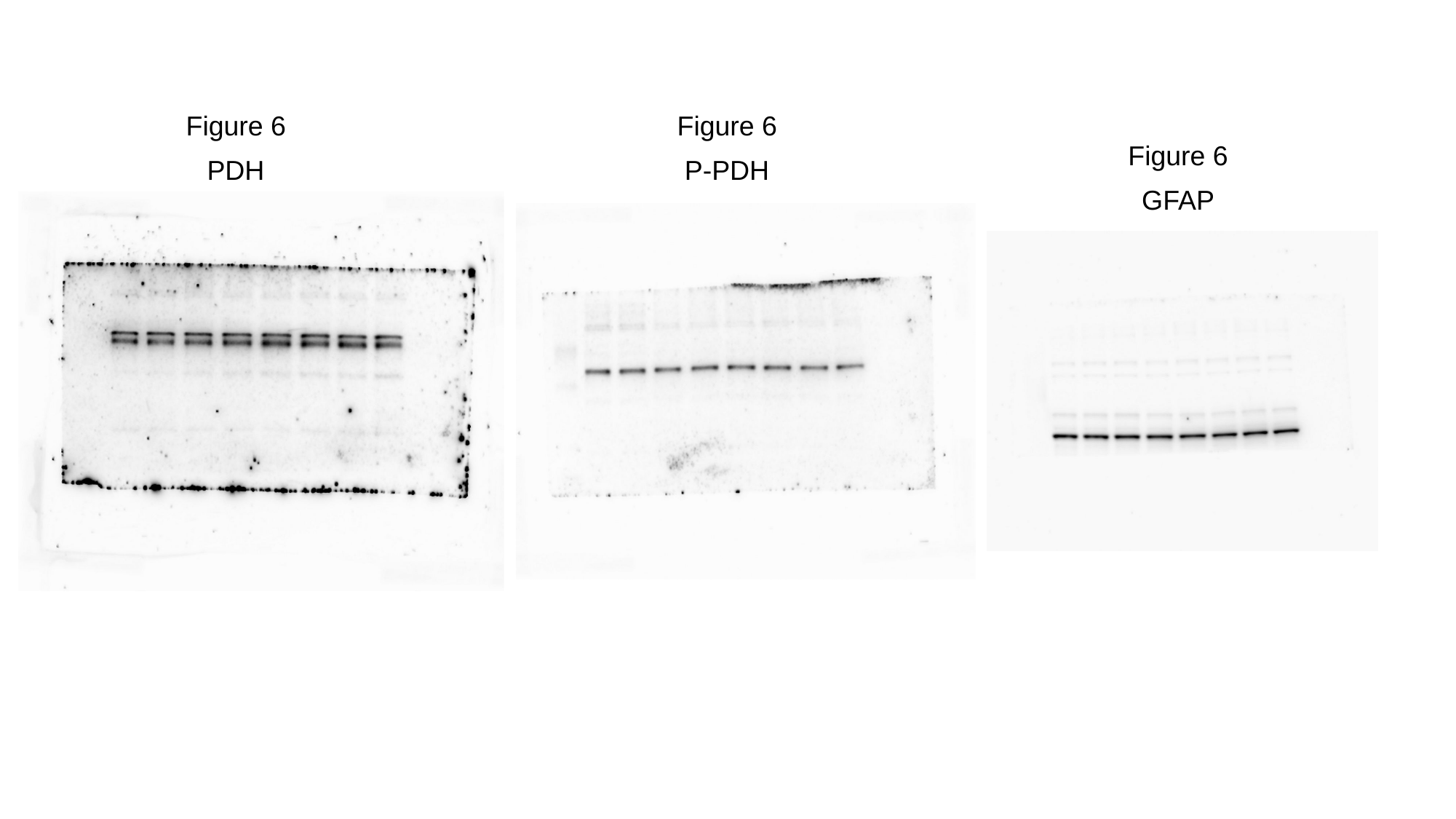

Figure 6
Figure 6
Figure 6
PDH
P-PDH
GFAP

## Slide 18
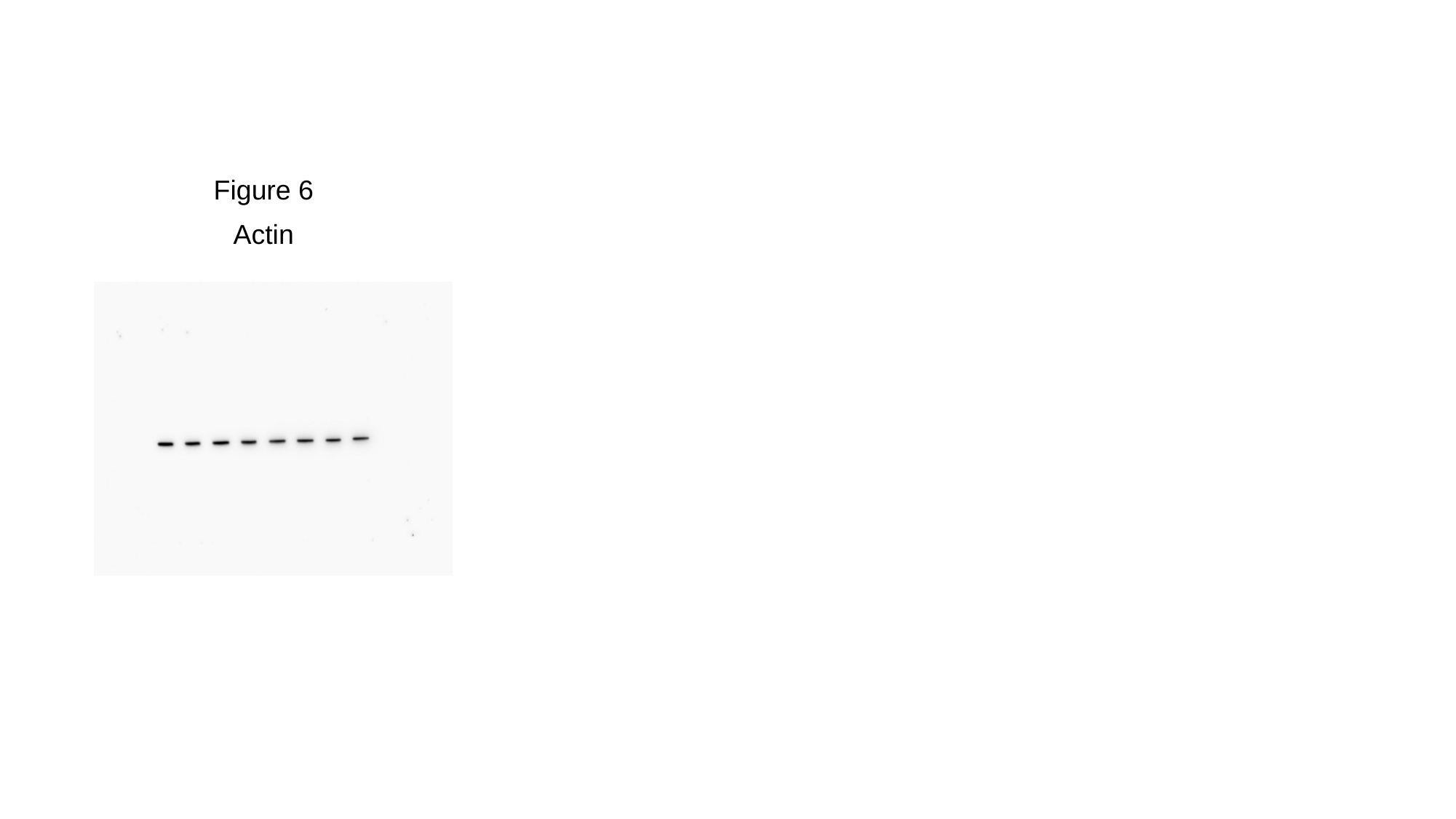

Figure 6
Actin
